# Friend or foe? Evolutionary history of glycoside hydrolase family 32 genes encoding for sucrolytic activity in fungi and its implications for plant-fungal symbioses

**DOI:** 10.1186/1471-2148-9-148

**Published:** 2009-06-30

**Authors:** Jeri Lynn Parrent, Timothy Y James, Rimvydas Vasaitis, Andrew FS Taylor

**Affiliations:** 1 Department of Forest Mycology and Pathology, Swedish University of Agricultural Sciences, Box 7026, Ulls väg 26a, SE 750 07 Uppsala, Sweden; 2 Department of Integrative Biology, University of Guelph, Guelph, Ontario, N1G 2W1, Canada; 3 Current address: Department of Ecology and Evolutionary Biology, University of Michigan, Kraus Natural Science Building, 830 North University, Ann Arbor, MI 48109-1048, USA; 4 Current address: Macaulay Institute, Craigiebuckler, Aberdeen, AB158QH, UK

## Abstract

**Background:**

Many fungi are obligate biotrophs of plants, growing in live plant tissues, gaining direct access to recently photosynthesized carbon. Photosynthate within plants is transported from source to sink tissues as sucrose, which is hydrolyzed by plant glycosyl hydrolase family 32 enzymes (GH32) into its constituent monosaccharides to meet plant cellular demands. A number of plant pathogenic fungi also use GH32 enzymes to access plant-derived sucrose, but less is known about the sucrose utilization ability of mutualistic and commensal plant biotrophic fungi, such as mycorrhizal and endophytic fungi. The aim of this study was to explore the distribution and abundance of GH32 genes in fungi to understand how sucrose utilization is structured within and among major ecological guilds and evolutionary lineages. Using bioinformatic and PCR-based analyses, we tested for GH32 gene presence in all available fungal genomes and an additional 149 species representing a broad phylogenetic and ecological range of biotrophic fungi.

**Results:**

We detected 9 lineages of GH32 genes in fungi, 4 of which we describe for the first time. GH32 gene number in fungal genomes ranged from 0–12. Ancestral state reconstruction of GH32 gene abundance showed a strong correlation with nutritional mode, and gene family expansion was observed in several clades of pathogenic filamentous Ascomycota species. GH32 gene number was negatively correlated with animal pathogenicity and positively correlated with plant biotrophy, with the notable exception of mycorrhizal taxa. Few mycorrhizal species were found to have GH32 genes as compared to other guilds of plant-associated fungi, such as pathogens, endophytes and lichen-forming fungi. GH32 genes were also more prevalent in the Ascomycota than in the Basidiomycota.

**Conclusion:**

We found a strong signature of both ecological strategy and phylogeny on GH32 gene number in fungi. These data suggest that plant biotrophic fungi exhibit a wide range of ability to access plant-synthesized sucrose. Endophytic fungi are more similar to plant pathogens in their possession of GH32 genes, whereas most genomes of mycorrhizal taxa lack GH32 genes. Reliance on plant GH32 enzyme activity for C acquisition in these symbionts supports earlier predictions of possible plant control over C allocation in the mycorrhizal symbiosis.

## Background

All Fungi are heterotrophic organisms, and the majority of fungal species rely solely upon plant tissues to meet their carbon (C) demands. In addition to saprotrophic fungi that decompose dead plant organic matter to acquire C, many fungi are biotrophs, forming intimate associations with living plant tissues. Associations between plants and biotrophic fungi are ubiquitous in nature; most plants are colonized from leaf to root with multiple fungal species. The interactions between biotrophic fungi and their plant hosts range from mutually beneficial (e.g. mycorrhizal associations) to context-dependent beneficial interactions (such as some endophytic fungi), to unilaterally antagonistic, potentially fatal pathogenic interactions.

In vascular plants, photosynthetically-derived C is delivered from source to sink tissues (e.g. roots) via phloem and sieve elements in the form of the non-reducing disaccharide sucrose [1]. Within plant sink tissues, sucrose is cleaved by extracellular invertase enzymes into equimolar concentrations of glucose and fructose. These monosaccharide molecules are then imported into plant cells via transport proteins and used either to meet cellular energy demands or as substrates for synthesizing other carbohydrate-containing storage molecules. The amount of sucrose allocated to various sink tissues is driven in part by the capacity of sink tissues to store or metabolize the imported carbohydrates (sink strength) [2]. Thus, increased invertase enzyme activity and the resultant decline in sucrose concentrations in sink tissues is intimately tied to phloem unloading and carbon allocation in the plant [3,4].

Invertase enzymes belong to the glycoside hydrolase family 32 (GH32) group of carbohydrate active enzymes, where family membership is designated based upon amino acid sequence conservation [5]. GH32 is a polyspecific enzyme family comprised of genes encoding for invertase (β-fructofuranosidase; EC 3.2.1.26) activity, as well as inulinase (EC 3.2.1.7, EC 3.2.1.64, EC 3.2.1.80), levanase (EC 3.2.1.65), and particular fructosyltransferase (EC 2.4.1.99, EC 2.4.1.100) and fructosidase (EC 3.2.1.153, EC 3.2.1.154) activities [6]. GH32 enzymes catalyze hydrolysis of the glycosidic bonds of their target carbohydrate substrates, which include – depending upon the enzyme – sucrose, and various oligo- and polysaccharides such as the fructans levan and inulin. Overall, the majority of enzymes in the GH32 family are functionally designated as invertase [7]. Invertase targets the terminal β-2,1 fructosidic bonds found in sucrose, inulin and levan. Plants typically contain multiple genes encoding for invertase enzymes, some of which are secreted, and others which are expressed intracellularly [8]. Invertase and other GH32 enzymes are not restricted to plants but are also known from bacteria and fungi [7].

Given the abundance of sucrose in living plant tissues, possession of functional GH32 genes that facilitate sucrose utilization would be seemingly advantageous for plant biotrophic fungi. Fungal GH32 activity could enhance fungal growth in plant tissues where sucrose concentrations are high, such as in the phloem and the apoplastic spaces of source and sink tissues where phloem loading and unloading occurs, respectively. However, sucrose diversion from plants to fungi is clearly disadvantageous to plants if there is no mechanism by which plants can regulate the timing or amount of C acquired by their fungal partners. Indeed, sucrolytic activity by fungal GH32 enzymes has been implicated in the invasive growth of several plant pathogenic fungi. *Uromyces fabae*, a rust fungus infecting *Vicia faba*, has been shown to specifically up-regulate its invertase expression during the plant infection process [9]. Infection of *Hordeum distichum *leaves by another pathogenic fungal biotroph, *Puccinia hordeii*, has been shown to cause a significant reduction in sucrose and monosaccharide concentrations in the apoplast of these tissues [10]. Thus, GH32 genes have been documented in several pathogenic fungal biotrophs and appear to be expressed in order to enhance C metabolism and access sucrose at infection sites. Given that a large number of plant-associated fungi are mutualistic (or commensal) rather than pathogenic, the expression of fungal invertase during some pathogen infection of plant tissues raises the important question: Do non-pathogenic symbiotic fungi also express invertase in their hosts?

Cultural studies of two fungal species that form mutualistic mycorrhizal associations, *Amanita muscaria *and *Hebeloma crustuliniforme*, showed that these species lacked invertase activity and failed to utilize sucrose as a C source in the absence of the plant host or host invertase enzyme [11]. Studies of beech mycorrhizas have also shown that monosaccharides rather than sucrose were the C compounds taken up by symbiotic fungal cells [12]. These results have led some investigators to hypothesize that all mycorrhizal fungi lack both a sucrose uptake system and the ability to metabolize sucrose via GH32 enzymes, and that they instead must rely upon the action of plant invertase enzymes to gain access to plant-derived C [13,14]. In contrast, results from several other studies provide evidence that suggests some mycorrhizal species may possess invertase [15,16], or may be able to grow on media with sucrose as a sole carbon source [17,18]. This disparity between results of different studies in conjunction with the fact that just a fraction of the phylogenetic and species diversity of mycorrhizal fungi have been examined for invertase activity (or sucrolytic abilities) currently limits our ability to draw general conclusions regarding whether mycorrhizal fungal genomes encode GH32 genes for sucrose utilization. Even less is known about these processes for other non-pathogenic biotrophic fungi, such as endophytic- and lichen-forming fungi [but see [19]].

To test the role of fungal GH32 genes in determining the mode of C acquisition and allocation in plant-fungal symbiosis we have investigated the evolution of GH32 genes throughout the fungi using a phylogenetic approach. Recent sequencing of over 75 fungal genomes has allowed us to determine the number of GH32 genes throughout the fungal kingdom and to trace the evolution of the gene family as it has evolved through numerous ecological transitions. Because the genomic data have largely emphasized plant and animal pathogenic fungi, we have also developed and implemented a PCR-based GH32 screening method for fungi. This approach allowed us to determine GH32 gene presence in obligately biotrophic fungi, many of which cannot be cultured (e.g., lichens and mycorrhizae, and to fill in the evolutionary and ecological gaps of the genome sequence data. By reconstructing the history of GH32 genes throughout fungi we addressed the following questions: 1) does GH32 gene presence and abundance positively correlate with a plant pathogenic ecological strategy; 2) do mycorrhizal, endophytic, and lichenic fungi typically possess or lack GH32 genes? Within plant-associated fungi we hypothesize that GH32 gene presence may be negatively correlated with the degree of mutualism and its presence or absence may therefore serve as a general marker for the sign of interaction (i.e., mutualistic vs. antagonistic) between a plant and a fungus.

## Results

### GH32 Gene phylogeny from fungal genome data

Seventy-six genomes from five fungal phyla were queried for GH32 gene presence: 9 Basidiomycota, 62 Ascomycota, 2 Zygomycota, 1 Chytridiomycota and 2 microsporidia. Forty-eight fungal genomes contained one or more GH32 gene sequences for a total of 130 GH32 sequences. The remaining 28 harbored no detectable GH32 (see Additional file 1). Nine sequences were manually edited to correct putative errors in their protein predictions, and an additional seven sequences were removed from the dataset entirely, due either to errors that could not be resolved or to redundancies in sequences retrieved by BLAST (See Additional file 2). The number of GH32 genes per genome ranges from 0–12. *Fusarium oxysporum *with 12 GH32 genes spanning 8 groups had the greatest number of genes of all fungi surveyed. It should be noted that these fungi vary in their degree of genome sequence coverage and this report of GH32 gene number must therefore be considered a minimum estimate until genome sequence efforts are completed.

**Figure 1:**
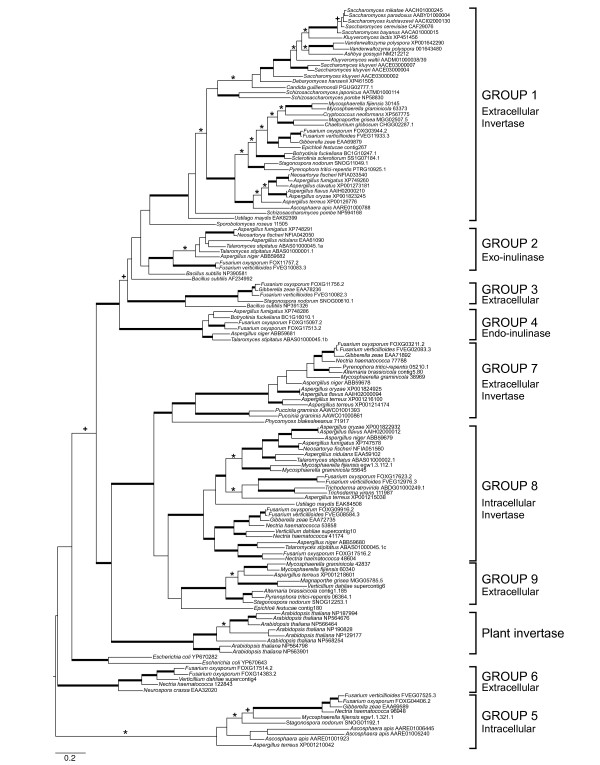
**Bayesian consensus phylogeny of the fungal glycoside hydrolase family 32 (GH32) gene family inferred from amino acid sequences retrieved from 76 fungal genomes**. Asterisks (*) indicate branches supported by Bayesian posterior probability ≥ 0.95, + symbols represent maximum likelihood bootstrap replicate frequencies ≥ 70%, and thickened branches represent support by both methods. Annotations of subcellular localization were predicted using SignalP v.3.0 [23] as shown for each of the 9 well-supported groups. For some clades there is no significant sequence homology to a GH32 gene encoding for a known function and only enzyme localization is predicted for these groups. GenBank accession numbers or sequence identifiers from genome projects are given for each sequence in the phylogeny. The databases queried for each genome sequence are indicated in Additional file 1.

Phylogenetic analysis of all 123 GH32 sequences from fungal genomes revealed a total of nine well-supported clades, four of which are previously unknown from fungi (Groups 3, 5, 6 and 9; Figure 1). Two sequences do not nest within any of the well-supported clades: the basidiomycete yeast, *Sporobolomyces roseus*, which is basal to group 1, and the Ascomycete grass endophyte, *Epichloë festucae*, placed sister to group 9. GH32 groups one and eight are the largest overall, containing 40 (32.5%) and 25 (20.3%) sequences, respectively. Group 1 harbors all of the Saccharomycetales and Schizosaccharomycetales sequences, and for this reason was referred to by Yuan *et al*. [20] as the yeast invertase clade, though both filamentous Ascomycete and Basidiomycete sequences are also found in this group. All of the other groups are comprised entirely of filamentous Ascomycete sequences except for group 7, which contains *Puccinia graminis *and *Phycomyces blakesleeanus *GH32 sequences, and group 8, which contains one GH32 sequence from the Basidiomycete smut fungus *Ustilago maydis*.

### Function and subcellular localization of GH32 groups

**Figure 2:**
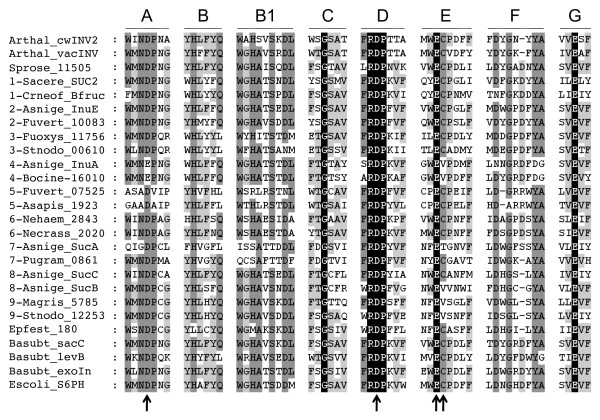
**Alignment of the conserved motifs of the Glycoside hydrolase family 32 (GH32) subfamilies as identified by previous researchers **[7,20]. Species are abbreviated by the following: Arthal = *Arabidopsis thaliana*, Asapis = *Ascosphaera apis*, Asnige = *Aspergillus niger*, Basubt = *Bacillus subtilis*, Bocine = *Botryotinia fuckeliana*, Crneof = *Cryptococcus neoformans*, Epfest = *Epichloë festucae*, Escoli = *Escherichia coli*, Fuoxys = *Fusarium oxysporum*, Fuvert = *Fusarium verticillioides*, Magris = *Magnaporthe grisea*, Necrass = *Neurospora crassa*, Nehaem = *Nectria haematococca*, Pugram = *Puccinia graminis*, Sacere = *Saccharomyces cerevisiae*, Sprose = *Sporobolomyces roseus*, Stnodo = *Stagonospora nodorum*. Two sequences of each clade are shown and indicated before taxon designation. Residues in black are completely conserved, residues in dark grey show 75% conservation, and residues in light grey show 50% conservation. Arrows indicate residues previously confirmed or suspected to be part of the active site [7,21].

All genome sequences presented in this study contain conserved motifs that suggest that they code for functional enzymes and are supported as being members of the GH32 gene family (Figure 2) [21]. Gene functions assigned to this group of carbohydrate active enzymes include invertase, inulinase, and fructosyl transferase activities; these enzymes can be expressed either intracellularly or secreted, and in some cases – as has been reported for the *S. cerevisiae *GH32 group 1 sequence – the same gene product may be expressed both intra- and extracellularly [22]. Groups 1,7 and 8 in the GH32 phylogeny contain sequences with known invertase function. Groups 2 and 4 sequence members exhibit inulinase activity and groups 3,5,8 and 9 cannot be assigned a specific function (Figure 1). Subcellular localization analysis performed using the programs SignalP v.3.0 [23] and CELLO v. 2.5 [24] concludes that groups 5 and 8 are expressed intracellularly, and the remaining clades are largely comprised of secreted enzymes.

### Fungal genome ancestral state reconstruction and phylogenetic independent contrasts

**Figure 3:**
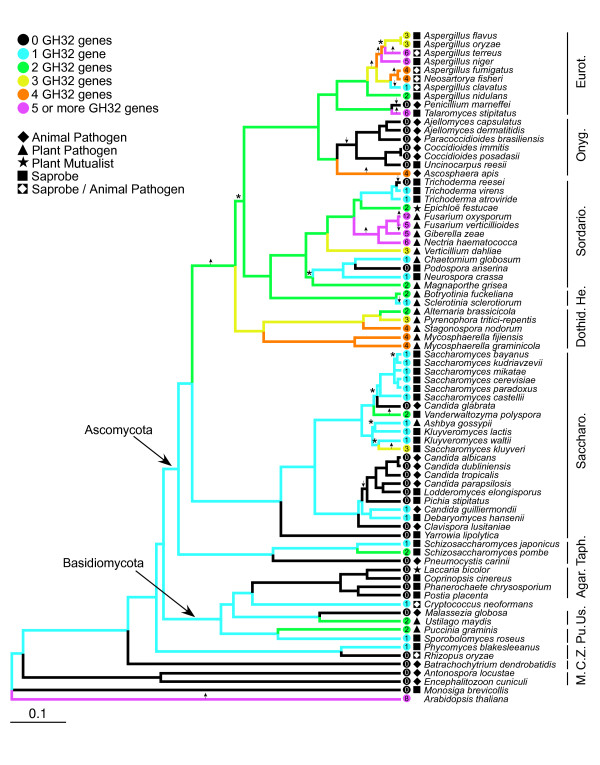
**Ancestral state reconstruction of Glycoside hydrolase family 32 (GH32) gene number across the phylogeny of sequenced fungal genomes**. Shown is a Bayesian consensus phylogeny inferred from RNA polymerase amino acid sequences, with molecular-clock like maximum likelihood branches calculated using TREE-PUZZLE v.5.3 [47]. All branches were supported with Bayesian posterior probabilities ≥ 0.95 except for the branch supporting the relationship between *Saccharomyces mikatae*, *S. bayanus *and *S. kudriavzevii*. The majority of branches were also supported by maximum likelihood bootstrap replicate frequencies ≥ 70%, those that were not supported are indicated with asterisk (*) symbols. Ancestral state reconstruction of GH32 gene number was computed from the clock-corrected phylogeny using the program CAFE v.2.0 [48]. Color of branches represents the predicted GH32 gene number for each ancestral node. GH32 gene numbers in extant taxa are shown in the colored circle to the right of the terminal branch of each species in the phylogeny. Ecological strategies of taxa are also illustrated to the right of each terminal branch (see figure legend for description of symbols corresponding to ecology). Arrows indicate branches for which the rate of gene gain (upward arrow) or gene loss (downward arrow) is significantly different. Clade classification is abbreviated by the following: Agar. = Agaricomycetes C. = Chytridiomycota, Dothid. = Dothideomycetes, Eurot. = Eurotiales, He. = Helotiales, M. = Microsporidia, Onyg. = Onygenales, P. = Pucciniomycotina, Saccharo. = Saccharomycetales, Sordario. = Sordariomycetes, Taph. = Taphrinamycotina, Us. = Ustilaginomycotina, Z. = Zygomycota.

The fungal phylogeny inferred from the six RNA polymerase genes yielded a well-resolved phylogeny with support for almost all nodes (Figure 3) and is consistent with recent phylogenetic hypotheses for fungi [25,26]. Ancestral state reconstruction of GH32 gene number across this fungal genome phylogeny predicted the most recent common ancestor of fungi after the divergence of microsporidia and Chytridiomycota possessed one GH32 gene. Fourteen independent losses of all GH32 genes were predicted, which included ancestral nodes of many of the lineages containing animal pathogens: microsporidia, the Onygenales (with the exception of the bee pathogen, *Ascosphaera apis*), and the genus *Candida*. The ancestor of the Agaricomycetes also is predicted to have lost GH32 genes. In contrast, gene family expansion was reconstructed for nodes leading to clades containing plant pathogens, the Eurotiales, Helotiales and Dothideomycetes. Significant changes between parent and descendent nodes in the rate of gene loss and duplication (λ) were detected on 7 and 12 branches respectively (Figure 3).

Phylogenetic independent contrast analysis complements the gene family expansion and contraction patterns observed in the ancestral state reconstruction, which shows a correlation between GH32 gene family size and ecological strategy. Both a positive correlation between GH32 gene number and plant pathogen status, and a negative correlation between animal pathogen status and GH32 gene number were statistically supported (Table 1). However, the result for animal pathogens was only statistically significant when taxa that are primarily saprotrophic and only facultatively pathogenic (*Neosartorya fisheri*, *Cryptococcus neoformans *and *Aspergillus *species) were not coded as pathogens. Correlation between GH32 gene number and saprotrophic status were positive but not statistically supported; mycorrhizal and endophytic fungal genomes are too few (one of each) to conduct a PIC analysis for these ecological strategies.

**Table 1:** Results of Phylogenetic Independent Contrasts (PIC) of ecology and invertase gene number calculated using Phylocom v.4.0.1b [49].

Ecological Strategy	MnConAll	SDConAll	N	*t*	df	P value
Plant Pathogen	1.621	1.735	8	2.6426	7	0.0333*
Saprotroph						
Saprotroph 1	0.171	1.827	20	0.4186	19	0.1710
Saprotroph 2	0.271	1.681	18	0.6840	17	0.5032
Animal Pathogen						
Animal path. 1	-0.748	1.971	15	1.4698	14	0.1637
Animal path. 2	-1.118	1.704	12	2.2728	11	0.0441*

PIC analysis was conducted for each ecological category with ecology treated as a binary state. For instances where species had ambiguous or multiple ecological states, separate analyses were run with the taxa in question coded both possible ways to determine the influence of the specific coding state for those taxa on the overall results. Saprotroph 1, *Cryptococcus neoformans *and *Aspergillus *spp. are coded as non-saprotrophic; Saprotroph 2,= *C. neoformans *and *Aspergillus *spp. are coded as saprotrophic; Animal path.1, *C. neoformans*, *Aspergillus *spp., and *Neosartorya fisheri *are coded as animal pathogens; Animal path. 2, *C. neoformans*, *Aspergillus *spp., and *Neosartorya fisheri *are coded as non-animal pathogens. MnConAll, average magnitude of independent contrast. SDConAll, standard deviation of independent contrasts, N, number of contrasts. Asterisk (*) symbols indicate P values less than 0.05, which are considered statistically significant.

### GH32 gene distribution in experimentally assayed fungi

Given that plant symbiotic fungi are underrepresented in complete genome sequences, we used degenerate primers to examine the distribution of GH32 genes in a phylogenetically and ecologically diverse suite of symbiotic fungi, with a particular emphasis on mycorrhizal and Basidiomycete taxa. We successfully obtained GH32 sequences for taxa that had previously been reported to exhibit invertase activity, *Schizophyllum commune *[27], *Pycnoporus cinnabarinus *[28] and *Rhizoscyphus ericae *[16], as well as for taxa chosen as positive controls, giving us reasonable confidence in the utility of this approach and the primers we designed for this purpose. A total of 149 fungal taxa were tested in total: 7 lichenic, 9 endophytic, 51 mycorrhizal fungi, 13 plant pathogens, 1 animal pathogen and 57 saprotrophs (Table 2). Several additional taxa tested have multiple possible ecological strategies, 5 saprotroph/endophytic, 4 saprotroph/plant pathogen, and 2 endophytic/mycorrhizal. Overall, we detected GH32 genes in 39 (26.2%) of the 149 fungal taxa that were assayed, and in all but one case (2 sequences detected in *Rhizoscyphus ericae*), only one GH32 gene sequence was recovered (See Additional file 3). The majority of the sequences that we detected are members of the GH32 group 1 subfamily, although a small number of sequences belong to GH32 groups 5,6,8 and 9.

**Table 2:** Total number of taxa surveyed for glycoside hydrolase family 32 (GH32) gene presence, and the percentage in which one or more GH32 gene was detected.

**Ecology**	**No. of taxa tested**	**No. of taxa with GH32**	**% of taxa with GH32**
*Animal pathogen*			
Genome	19	2	11
Experimental	1	0	0
*Endophyte*			
Genome	1	1	100
Experimental	9	7	78
*Mycorrhizae*			
Genome	1	0	0
Experimental	51	3	6
*Plant Pathogen*			
Genome	17	17	100
Experimental	13	5	38.4
*Saprotroph*			
Genome	29	18	62
Experimental	57	19	33
*Lichen*			
Genome	-	-	-
Experimental	7	3	43
*Mycorrhizae/Endophyte*			
Genome	-	-	-
Experimental	2	2	100
*Saprotroph/Plant Pathogen*			
Genome	-	-	-
Experimental	4	0	0
*Saprotroph/Animal Pathogen*			
Genome	9	9	100
Experimental	-	-	-
*Saprotroph/Endophyte*			
Genome	-	-	-
Experimental	5	0	0
*Ascomycota*			
Genome	62	42	68
Experimental	46	22	48
*Basidiomycota*			
Genome	9	4	49
Experimental	103	17	16.5
*Total*			
Genome	76	47	62
Experimental	149	39	26.2

Data are categorized by Ecology, and a separate tally is made of sequences belonging to the phylum Ascomycota or Basidiomycota. Each category is further subdivided to differentiate data retrieved from genome databases (Genome) from that assayed by PCR (Experimental).

Both phylogeny and ecological strategy are important predictors of GH32 presence. Overall, GH32 sequences were detected with significantly greater frequency in Ascomycete taxa (22/46 = 48%) than in Basidiomycete taxa (17/103 = 17%) (one-tailed Fisher's exact test: probability Ascomycota GH32 presence greater than Basidiomycota: p < 0.001). Similar to the results found with fungal genome data, the animal pathogen taxa did not possess GH32 genes, while they were found in plant pathogens, though at a lower detection frequency than the genome data (5/13 = 38.4% PCR assay results vs. 100% detection in fungal genome data). GH32 genes were detected in the majority of endophytic fungi (7/9 = 77.8%), and in nearly half of the lichenic fungi (3/7 = 42.9%) as well as many saprotrophs (19/57 = 33.3%). In contrast, of the 51 mycorrhizal and 2 mycorrhizal/endophytic fungal taxa surveyed, GH32 genes were detected in only 5 species: *Sebacina incrustans*, *Elaphomyces *cf. *verruculosus*, *Rhizoscyphus ericae*, *Meliniomyces bicolor *and *Phialophora finlandica*, of which only one (*S. incrustans*) is a member of the phylum Basidiomycota (Class Agaricomycetes), and the rest are Ascomycota species. While only one mycorrhizal Agaricomycetes species possessed a GH32 gene, a number of the saprotrophic Agaricomycetes taxa tested positive for GH32 gene presence, suggesting that the paucity of GH32 genes found in mycorrhizal Agaricomycetes is not due to the wholesale absence of the gene family in this clade.

**Figure 4:**
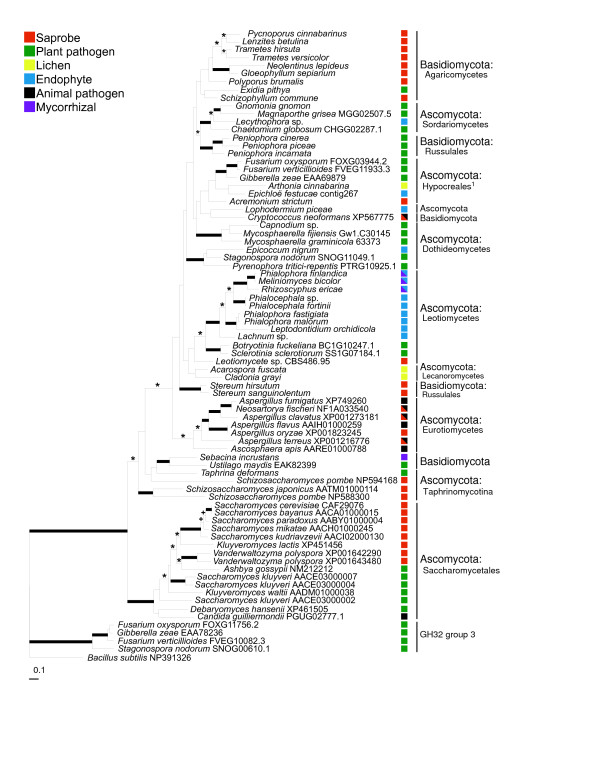
**Bayesian consensus phylogeny of the Glycoside hydrolase family 32 (GH32) sub-group 1, which encodes for an extracellular invertase enzyme, inferred from nucleotide sequences recovered from either fungal genome searches or by PCR and sequencing**. Asterisks (*) indicate support by Bayesian posterior probability = 0.95, + symbols represent support by maximum likelihood bootstrap replicate frequencies = 70%, and thickened branches represent support by both methods. Colored squares adjacent to terminal branches indicate the ecological strategy of each fungal species (see figure legend for the correspondence between coloration and fungal strategy). ^1 ^This clade contains one sequence, *Arthonia cinnabarina*, that is not a member of the Hypocreales.

The Bayesian GH32 group 1 phylogeny inferred from nucleotide sequence data demonstrated the placement of the new GH32 sequences acquired by PCR and sequencing among those retrieved from fungal genomes. A number of sequences were too short to include in the analysis, and the relatively short length of the sequenced fragments overall is likely responsible for the lack of statistical support for a number of branches (Figure 4). The GH32 group 1 gene phylogeny is markedly different than the species phylogeny. For example, Basidiomycota sequences are found in five separate positions in the phylogeny, each nested within Ascomycota. The Polyporales-derived sequences form a clade, but the multiple sequences from Russulales (*Stereum *and *Peniophora*) do not form a clade. *Cryptococcus neoformans *groups sister to *Lophodermium piceae *within a clade of filamentous Ascomycota (Figures 1 &3), although posterior probabilities and maximum likelihood bootstrap support for the placement of *C. neoformans *are less than 95% and 70%, respectively.

## Discussion

Sucrose is the primary compound used by most plants to transport carbon throughout their tissues, and its abundance within plants makes it a valuable carbon source for the many fungi that are obligate plant associates. In order to directly utilize sucrose, fungi must possess the necessary enzymes to cleave sucrose into its constituent monosaccharides. The aim of this study was to explore the distribution and abundance of GH32 genes that encode for sucrolytic activity in fungi in order to understand how the potential for sucrose utilization is structured within and among major ecological guilds and evolutionary lineages. Using a combination of bioinformatics and PCR-based assays targeting the breadth of fungal phylogenetic and ecological diversity, we detected a total of nine well-supported subfamilies of fungal GH32 genes. The number of GH32 genes recovered from an individual species ranged from 0–12, with a mean value of 1.62 and 0.27 copies per taxon detected in the genome and experimental surveys, respectively. Two of the nine GH32 groups found in fungi contained the majority of the sequences: group 1, a secreted invertase enzyme, and group 8, an intracellular enzyme of putative invertase function [20]. PIC analysis of the fungal genome data showed GH32 gene abundance was significantly correlated with ecological strategy. GH32 abundance was negatively correlated with animal pathogenic fungi and positively correlated with plant biotrophic fungi – plant pathogens, endophytic and lichenic fungi – with the notable exception of mycorrhizal fungal taxa. Few mycorrhizal fungal species were found to have GH32 genes when compared to other plant-associated fungi, only one of which belonged to the phylum Basidiomycota. We also observed a phylogenetic signal in GH32 distribution among fungi, with greater GH32 gene prevalence found in the Ascomycota than in the Basidiomycota. In the following paragraphs we will consider the significance of GH32 distribution and abundance patterns for the evolutionary history of this gene family in fungi, the functional diversity both within and between ecological guilds of fungi, and its relevance for the sign and strength of ecological outcomes in plant-fungal interactions.

### Functional diversity within and among fungal ecological guilds

Ecological strategy was an important predictor of both the presence and abundance of GH32 genes. At the coarsest level, there is a distinct difference in GH32 distribution between animal and plant-associated fungi, with the former generally lacking, and the latter generally possessing GH32 genes. It is logical that animal associates would lack sucrolytic capabilities because sucrose is neither synthesized by animals nor is it stored in their tissues. One notable exception is the presence of GH32 genes in *Ascosphaera apis*, the causal agent of chalkbrood disease of honeybees, which can grow in the sucrose-rich environment of honeycombs and infects developing honeybee larvae [29]. A more refined view of plant-associated fungi also reveals distinctions among different ecological strategies within this more general classification. We detected GH32 genes in all plant pathogens for which whole genome sequences were available, and in 38% of those surveyed by PCR. They were also found in the majority (78%) of endophytic fungi and a number (43%) of lichen-forming fungi (Table 2). GH32 genes were not only present in all plant pathogen genome sequences, but GH32 gene family size was also found to be expanding in this group, a pattern that has been shown for many gene families in plant pathogenic Ascomycota genomes [30]. In contrast, GH32 genes were absent in the only currently completed mycorrhizal fungal genome, *Laccaria bicolor *[31], and in the vast majority of the mycorrhizal taxa that we tested experimentally, particularly those belonging to the Basidiomycota.

While general trends were identified among ecological guilds of fungi, variation within guilds was also detected, highlighting potential functional diversity in C acquisition strategies harbored within an ecological guild, and plasticity of ecological strategy within individual taxa depending upon host identity or environmental conditions. Although GH32 genes were rare in mycorrhizal fungi, they were not entirely absent. We found putatively functional GH32 gene copies in 5 of the 53 mycorrhizal taxa tested (9.4%): Four of the five taxa contained at least one gene copy from the secreted invertase group (GH32 group 1; Figure 1); *E. cf. verruculosus *contained a copy from GH32 group 8, an intracellularly-expressed putative invertase, and *R. ericae *contained both a GH32 group 1 gene and a second GH32 gene from group 5, an intracellularly-expressed gene of uncertain function. While gene presence is not equivalent with enzyme activity, the presence of conserved motifs in these sequences, combined with data from pure culture studies documenting invertase activity in *R. ericae *[16], provide good evidence that sucrose utilization facilitated by invertase activity is possible for this subset of mycorrhizal taxa. Several of these species are also plastic in their ecological strategies, forming ectomycorrhizal, ericoid mycorrhizal and endophytic associations (*M. bicolor*, *P. finlandica*). If these GH32 genes are functional in these species, what is the timing and location of their expression? Are they expressed within symbiotic tissues to increase C acquisition, and are they expressed during each of the possible ecological strategies? In the case of *E. cf. verruculosus *and *R. ericae*, which possess intracellular GH32 genes, do these taxa possess sucrose import systems? These remain open questions, but answering them will provide additional insight into the functional similarities and differences in C interactions among different plant-fungal symbioses, and could expand our model of C transfer in the ectomycorrhizal symbiosis, which currently assumes sucrose import by fungi is not possible, and that sucrose hydrolysis is carried out solely by the plant partner [32,33].

For species with uncertain or variable ecologies, GH32 gene presence may serve as an indicator of potential endophytic ability or biotrophic growth, as illustrated by the "wood-decay" species found to possess GH32 genes (Figure 4). While commonly thought of as saprotrophic, the wood decay fungi *Schizophyllum commune*, *Pycnoporus *spp., and several Polyporales species that tested positive for GH32 presence can also grow endophytically inside attached limbs of temperate oak trees [34], and stems of *Theobroma cacao *[35,36] and *Elaeis guineensis *[36]. Other endophyte surveys have also cultured members of the Basidiomycota from woody tissues such as the inner bark [35], and from conifer fine roots, which in some cases can exhibit a growth pattern and mantle morphology similar to that of ectomycorrhizal fungi [37]. Phloem-containing tissues may select for those wood-decaying endophytes that can metabolize both cellulose and sucrose, and GH32 activity may play an important role in facilitating the establishment of these early-successional wood-decay fungi.

### Evolutionary history of GH32 genes in the Basidiomycota

Ancestral state reconstruction of GH32 gene numbers across the fungal genome phylogeny predicts 14 separate losses of GH32 genes. Many of these losses are at nodes leading to terminal taxa that are obligate animal pathogens, but complete GH32 gene loss is also predicted for the most recent common ancestor of the Agaricomycete lineage (Figure 3). Using PCR assays we detected GH32 genes in only one of the 46 mycorrhizal Agaricomycetes taxa tested, *Sebacina incrustans*, which is a member of the order Sebacinales, the most basal clade of Agaricomycetes [38]. GH32 genes were also less commonly detected in non-mycorrhizal Agaricomycetes than in Ascomycota taxa, though they were not entirely absent. GH32 genes were detected in 30% (16 of 54 surveyed) of non-mycorrhizal Agaricomycetes belonging to four orders: Agaricales, Auriculariales, Polyporales and Russulales. These species were either saprotrophs, particularly those thought to be associated with wood decay, such as *Pycnoporus cinnabarinus *and *Fomitopsis pinicola *and the three *Peniophora *species, or plant pathogens, such as *Exidia pithya*. GH32 sequences from these Basidiomycota classes did not form a monophyletic group in the GH32 group 1 phylogeny, and instead exhibited a punctate distribution across the gene phylogeny, possibly due to the difficulty of reconstructing the phylogeny because of the small size of the sequenced fragment or due to multiple independent gains by horizontal transfer. Regardless of the mechanism, the possession of GH32 genes in the genome of Agaricomycetes is a rapidly evolving trait.

### Comparisons between experimental data and genome data

One caveat to this research is that with incomplete genome sequence data and degenerate PCR-based assays, the estimates of GH32 gene number are merely lower estimates; additional copies may exist that were not detected. In the case of the genome data, although we only included genomes that had been sequenced to a reasonable level of coverage, until sequencing is complete it is possible that the actual number of GH32 genes in these genomes may differ from what we report here. When we compare the frequency of GH32 gene presence between the genome and experimental data separately for the Ascomycota and Basidiomycota data, we find that the frequency of GH32 gene occurrence is lower in the experimental data than in the genome data, though these differences are not statistically significant (one-tailed Fisher's exact test, probability genome greater than experimental Ascomycota p = 0.1018; probability genome greater than experimental for Basidiomycota p = 0.0697). This result is likely due to the fact that PCR-based assays will suffer from false negatives due to PCR bias, and this approach will never yield as complete of a picture as the entire genome data will, even in cases where care has been taken to design and test many degenerate primer pairs. In order to maximize gene detection, primers were anchored in conserved gene regions, tailored to specific GH32 genes and phylogenetic groups, and tested extensively in all possible combinations. Those primer pairs ultimately chosen worked successfully across a wide range of taxa, and we were able to amplify the genes in taxa for which invertase enzyme activity had previously been reported, such as *Schizophyllum commune *[27] and *Rhizoscyphus ericae *[16]. Nonetheless, PCR-bias likely explains why fewer gene copies were detected relative to the genome data, particularly for GH32 gene copies outside of group 1. However, in light of this limitation, there are a number of additional lines of evidence that give us confidence in our interpretation of the results with respect to the implications for fungal ecology.

Comparisons between the genome data and the experimental data show the same patterns of GH32 gene frequency among phylogenetic groups and ecological guilds. For example, statistical analysis of the Ascomycota genome data reveals significant differences in GH32 presence among ecological strategies, such that GH32 occurrence is greatest in plant pathogens, lowest in animal pathogens and intermediate in saprotrophs (ChiSquare likelihood ratio test = 24.464, p < 0.001). This pattern is mirrored in the experimental data (the greatest GH32 gene occurrence in Ascomycota was found in plant pathogens, the lowest in animal pathogens and intermediate in saprotrophs), though this result lacks statistical significance. Similarly, both the genome data and the experimental data show that there is lower GH32 occurrence in the Basidiomycota taxa relative to Ascomycota taxa, though this result is statistically significant only in the experimental data. Taken together, our ability to detect GH32 presence in taxa which have been determined to possess invertase activity, as well as the consistency in the patterns between the genome data and experimental data give us confidence in our evaluation of GH32 gene presence in ecological guilds for which genome data are currently unavailable or extremely sparse, such as for mycorrhizal fungi in the Agaricomycetes, and lichenic and endophytic fungi in the Ascomycota.

## Conclusion

This study analyzed the distribution of GH32 genes from fungi to determine if there is a relationship between sucrolytic capability and ecological niche in plant symbiotic fungi. A strong signature of both ecological strategy and species phylogeny on GH32 gene number was determined based on data mining of complete genomes. Extensive gene duplications of GH32 are observed in several groups of filamentous Ascomycota such as *Fusarium *and *Aspergillus*. Phylogenetic reconstructions showed that expansions of GH32 genes coincided with switches to a plant pathogenic habitat, and conversely loss of all GH32 genes was observed on branches leading to nearly all animal pathogens. Experimental results using PCR targeting GH32 homologues from a diversity of plant-associated fungi found GH32 genes in the secreted subfamily 1 to be the most phylogenetically widespread.

We report for the first time the sequences of GH32 genes from endophytic, lichenic, and mycorrhizal fungi, highlighting the potential for functional diversity within these ecological guilds. GH32 genes were almost entirely absent among the large number of Basidiomycota ectomycorhizal fungal species tested (1 out of 46). Reliance on plant GH32 enzyme activity for C acquisition in these symbionts supports earlier predictions of a general absence of invertase in mycorrhizal fungi [11], and a highly evolved mutualistic relationship between plants and mycorrhizal fungi [17], a remarkable scenario in light of the high degree of phylogenetic diversity spanned by mycorrhizal fungal taxa. Whether the plant host is able to detect fungal invertase activity and use such a signal to differentiate antagonistic from mutualistic biotrophic symbionts is a completely speculative, though plausible hypothesis. Additional experiments using gene disruption mutants to investigate plant response to fungal GH32 expressed in symbiotic tissues will be an important step in clarifying the role fungal GH32 genes play in a plant's ability to distinguish friend from foe.

## Methods

### Database Searches

An initial database search was performed using the *Saccharomyces cerevisiae *GH32 amino acid sequence (GenBank accession number CAF29076) and all fungal sequences were obtained that showed significant homology (significance score of e^-5 ^or lower) to the GH32 query gene. A preliminary phylogeny was generated, and the resulting phylogeny was compared to that reported in [20] to confirm that all of the unique clades of fungal GH32 genes that have been identified to date were represented. From this initial search eight sequences were selected that spanned the fungal GH32 gene phylogeny (query sequences and accession/gene model numbers: *S. cerevisiae*, CAF29076; *Fusarium verticillioides*, FVEG10083.3, FVEG10082.3; *Botryotinia fuckeliana*, BCIG16010.1; *Aspergillus terreus*, XP001215038; *Stagonospora nodorum*, SNOG01192.1; *Sporobolomyces roseus *11505; *Neurospora crassa*, EAA32020), and used as query sequences to retrieve homologous sequences from all fungal genome databases available as of 1 May, 2008, using either blastp or tblastn [39] search algorithms. Three *Bacillus subtilis *and two *Escherichia coli *bacterial sequences with significant sequence homology to the query sequences were also identified and included in the analysis. All fungal taxa and databases queried in this study are listed in Additional file 1.

When predicted gene models were unavailable, they were manually predicted by removal of introns and three-frame translation. In cases where proteins were incorrectly predicted, or where sequences were truncated because they spanned multiple contigs, full-length sequences were reconstructed and manually annotated. A small number of sequences that showed significant homology to the query sequences were either unalignable, contained model prediction errors that could not be confidently corrected, or possessed multiple model predictions, and were therefore removed from the dataset prior to analysis (indicated in Additional file 2).

In order to estimate a fungal phylogeny of the taxa for which complete genome sequencing has been accomplished, RNA polymerase (RP) genes were retrieved from available fungal genomes, the choanoflagellate *Monosiga brevicollis*, and the plant *Arabidopsis thaliana *by blast using the six *Saccharomyces cerevisiae *RP genes (RPA1, RPA2, RPB1, RPB2, RPC1, and RPC2) as query sequences (GenBank Accession nos. P10964.2, P22138.1, P04050.2, P08518.2, P04051.1, P22276.2). Additional file 4 lists accession numbers for all RP genes.

### Molecular Methods

Due to the uneven distribution of currently available fungal genomes across the phylogenetic and ecological diversity of fungi, and because many symbiotic taxa are difficult to culture, we used a PCR-sequencing approach to detect GH32 gene presence and evaluate correlations between phylogeny, ecology and GH32 distribution. A suite of degenerate primers was designed to target each unique clade of the GH32 gene phylogeny inferred from the fungal genome data (primer sequences are listed in Table 3). A phylogenetically diverse set of fungi containing multiple representatives of the major fungal ecological strategies – saprotrophs, mycorrhizal fungi, endophytic fungi, lichenic fungi, plant pathogens and animal pathogens – were obtained as either cultures, vouchered fruiting body specimens, or DNA extracts and are listed in Additional file 3. Cultures were grown in Petri dishes containing either MMN, MEA or PDA media, depending on species-specific requirements, at 24°C in the dark, atop a thin layer of cellophane. Mycelia was harvested from the cellophane surface and stored in 2× CTAB at -20°C pending DNA extraction. DNA was extracted from fruiting bodies or fungal mycelia using the protocol of Vilgalys and Hester [40]. Culture identity was confirmed by sequencing the ITS rRNA gene region.

**Table 3:** List of PCR primers designed to assay fungi for glycoside hydrolase family 32 (GH32) gene presence.

**Primer**	**Primer sequence 5'→3'**	**Direction**	**Length**	**Tm°C**
INV1-1F	TTYATGAAYGAYCCNAAYGG	Forward	20	53.96
INV1-FA1F	CARCAYTGGGGNCAYGCNAC	Forward	20	62.76
INV1-B1F	GGNAAYCARCAYTGGGGNCAYGC	Forward	23	67.97
INV2-1F	AACTGGATSAAYGAYCCNAAYGG	Forward	23	59.6
INV4-2F	TTYTTYCARCAYAAYCCNAC	Forward	20	50.74
INV5A-1F	GGITGGHTIAAYGAYCCNTGYG	Forward	22	61.88
INV5B-A1F	GGICRIATYGGNGAYCCNTG	Forward	20	58.39
INV5B-B1F	GGITGGATGAAYGAYCCNATG	Forward	21	60.57
INV5B-B1.1F	GGITGGATGAAYGAYCC	Forward	17	55.65
INV5C-1F	GGITGGMTGAAYGAYCCTTG	Forward	20	61.74
INV6-1F	TGYCCIGARTGYYCNGA	Forward	17	52.34
INV1-1R	ACYTTIGGRTCNCKRAA	Reverse	17	46.05
INV1-B1R	TTNARNGTRTARATNGCNACIACICC	Reverse	26	55.76
INV-1-B2R	GGRAARAANCCNGAIGTRTT	Reverse	18	52.11
INV1-2R	TAITICCARTTNGANGCCCA	Reverse	20	62.52
INV1-2.1R	TAITICCARTTRTTNGCCCA	Reverse	20	61.54
INV2-2R	GGICCICCIGGRTTNAGNCC	Reverse	20	61.29
INV4-1R	TCIGGIACYTCCCANCC	Reverse	17	55.45
INV5A-1R	RCAICCIGTRAANACNCC	Reverse	18	49.03
INV5B-1.1R	CRTAIGGRTCNCGRAANGC	Reverse	19	54.69
INV5B-1.3R	CRTAIGGRTCNCGRAANCC	Reverse	19	53.52
INV5B-1.4R	CRAAIGGRTCYCTRAANCC	Reverse	19	50.09
INV5B-1.5R	CRAAIGGRTCNCGRAANCC	Reverse	19	53.52
INV5C-1R	GTGAYGTTCCANCCYTCNGGNGG	Reverse	23	68.6
INV6-2R	CAIGAYTTIGCNGCRTACCA	Reverse	20	61.22

Primer pair combinations and PCR conditions that correspond to a particular GH32 sequence are given in Additional file 3. Tm, melting temperature.

PCR amplification of GH32 genes was performed in 25 μl reactions with approximately 10 ng of template DNA, using 0.875 units Thermo Red Taq DNA polymerase with the supplied PCR buffer S (Saveen & Werner) under the following conditions: initial denaturation at 94°C for 7 minutes, followed by 35 cycles at 94°C for 45 s, 45–56°C for 30 s depending upon the primer set used (see Table 3 for primer pair-specific annealing temperatures), 72°C for 60 s, and a final extension at 72°C for 10 min. PCR products containing multiple bands, of which at least one was the expected size for the primer set used, were ligated into the PCR 2.1-TOPO vector and transformed into chemically competent *Escherichia coli *strain TOP 10 cells according to manufacturer's protocols (Invitrogen, Carlsbad, CA). Eight colonies from each cloning reaction were screened by PCR and 1–3 fragments of the appropriate size were purified using QIAaquick PCR purification kit (Qiagen, GmbH, Germany), sequenced using Big Dye chemistry v. 3.1 and visualized on an ABI3730 automated sequencer (Applied Biosystems, Foster City, CA). For PCR products that yielded a single band of the expected size, cloning was omitted and products were purified and directly sequenced. All GH32 sequences generated by PCR have been deposited in GenBank with accession numbers GQ277570–GQ277609.

### Sequence alignments and phylogenetic analyses

GH32 and RP amino acid sequences from fungal genome data, and the GH32 group 1 nucleotide sequences from genome and experimental data, were initially aligned using MUSCLE [41], and the resulting alignments were manually edited. Ambiguous or unalignable sequence regions were excluded from the analyses. Amino acid alignments were analyzed using ProtTest v. 1.4 software [42] and the GH32 group 1 nucleotide alignment was analyzed with MrModeltest v2.3 [43] using the Akaike information criterion (AIC) to select the best-fitting model of evolution for each dataset.

Phylogenies were estimated using MrBayes version 3.1.2 [44]. The GH32 and RP protein datasets were both analyzed using two independent runs of 10 × 10^6 ^and 2 × 10^6 ^generations of four chains, respectively, each with the WAG amino acid model, gamma distributed substitution rates across variable sites and an estimated proportion of invariant sites. Trees sampled in the first 1 × 10^6 ^generations were discarded (burn-in) and the remaining trees were used to compute the consensus of the sampled trees, the posterior probabilities of clades, and average branch lengths. Bayesian analysis of the GH32 Group 1 nucleotide dataset was conducted with two independent runs for 2.5 × 10^6 ^generations with four chains, an initial burn-in of 1 × 10^6 ^generations using the GTR nucleotide model, gamma distributed substitution rates and an estimated proportion of invariant sites. Run convergence was assessed by plotting ln L using the software program Tracer v1.4 [45]. Support for nodes was also assessed for all datasets using maximum likelihood bootstrap (1000 replicates) as implemented in PHYML [46]. Molecular clock-like maximum likelihood branch lengths were also calculated for the RP Bayesian consensus phylogeny using TREE-PUZZLE v.5.3 [47], for subsequent use in the ancestral state reconstruction analyses.

### Fungal genome ancestral state reconstruction and phylogenetically independent contrasts

Reconstruction of ancestral GH32 gene copy number for the fungal genome phylogeny was conducted with the program CAFE v.2.0 [48] using the Bayesian consensus RP phylogeny with clock-like maximum likelihood branch lengths and the number of GH32 genes as input. CAFE models gene gain and loss across a given phylogeny as a stochastic birth and death process governed by a rate parameter (λ) that is allowed to evolve across the phylogeny. CAFE also calculates the maximum likelihood solution for the number of gene copies at ancestral nodes and tests for significant changes in λ using Monte Carlo re-sampling.

Phylogenetic independent contrast (PIC) analysis was used to test for correlations between ecology and GH32 distribution across the fungal genome phylogeny using the program Phylocom v.4.0.1b [49]. However, PIC as implemented in Phylocom and similar programs cannot analyze a multistate unordered categorical variable such as the ecological strategy variable in this dataset. To overcome this limitation, the ecological strategies were treated as binary data, and comparisons were then made between the continuous data (GH32 gene number) and the binary trait of each ecological strategy (e.g., plant pathogen or not). This analysis was then repeated for each of the ecological strategies coded in the dataset. To deal with taxa with ambiguous ecological strategies or those that could be coded in multiple ways, such as *Cryptococcus neoformans*, which can cause disease in immune-compromised patients (animal pathogen coding), but exists in nature primarily as a saprotroph (saprotroph coding), analyses were repeated with these taxa coded as one state or another, to examine the influence of their coding on the outcome of the analyses. For each PIC analysis, one sample, two-tailed t-tests were performed to evaluate the statistical significance of the average magnitude of independent contrasts across all contrasts, where sample size (N) used in the statistical analysis is the number of contrasts.

## Authors' contributions

JLP contributed to all aspects of this research, TYJ assisted with phylogenetic analysis and drafting the manuscript, RV isolated fungi used in the PCR assays and critically revised the manuscript, AFST assisted in project development and drafting the manuscript. All authors have read and approved of the final manuscript.

## Supplementary Material

Additional file 1 **List of fungal genomes used in this study**. This table includes the classification, ecological affiliation and the number of GH32 genes detected for each fungal genome queried. Click here for file

Additional file 2 **List of GH32 genes detected in fungal genomes**. Table includes the number of GH32 genes detected, the GH32 clade to which each gene belongs, the accession number of each sequence, and the database from which they were retrieved. Click here for file

Additional file 3 **List of fungal taxa tested for GH32 gene presence**. The table includes the classification and ecological guild of each fungal taxon assayed for GH32 genes, whether or not they were detected, and the primer set(s) and PCR conditions used to assay each taxon. Click here for file

Additional file 4 **List of fungal genome RNA polymerase accession numbers**. This table includes information for the data used for phylogenetic reconstruction of fungi with completely sequenced genomes. Click here for file
