# Supplementary material for: Friend or foe? Evolutionary history of glycoside hydrolase family 32 genes encoding for sucrolytic activity in fungi and its implications for plant-fungal symbioses
Source: BMC Evol Biol. 2009 Jun 30;9:148. doi: 10.1186/1471-2148-9-148 (PMC2728104; doi:10.1186/1471-2148-9-148)
Supplement: Additional file 1 — List of fungal genomes used in this study. This table includes the classification, ecological affiliation and the number of GH32 genes detected for each fungal genome queried. [file 1471-2148-9-148-S1.doc]

Additional file 1. List of fungal genomes used in this study, their taxonomic and ecological affiliation and the number of GH32 genes detected.

| **Organism** | **Classification** | **No. of GH32 genes** | **GH32 clade (gene no. in clade)** | **Ecological guild** | **Database Queried** |
| --- | --- | --- | --- | --- | --- |
|  | **Phylum Ascomycota** |  |  |  |  |
| *Ajellomyces capsulatus* | Pezizomycotina; Eurotiomycetes | 0 | 0 | Animal pathogen | NCBI |
| *Ajellomyces dermatitidis* | Pezizomycotina; Eurotiomycetes | 0 | 0 | Animal pathogen | Washington Univ. |
| *Alternaria brassicicola* | Pezizomycotina;Dothideomycetes | 2 | 7, 9 | Plant pathogen | Washington Univ. |
| *Ascosphaera apis* | Pezizomycotina; Eurotiomycetes | 4 | 1, 5(3) | Animal pathogen | USDA |
| *Ashbya gossypii* | Saccharomycotina;Saccharomycetes | 1 | 1 | Plant pathogen | NCBI |
| *Aspergillus clavatus* | Pezizomycotina; Eurotiomycetes | 1 | 1 | Animal pathogen | NCBI |
| *Aspergillus flavus* | Pezizomycotina; Eurotiomycetes | 3 | 1, 7, 8 | Saprotroph/  Plant pathogen/  Animal pathogen | TIGR |
| *Aspergillus fumigatus* | Pezizomycotina; Eurotiomycetes | 4 | 1, 2, 4, 8 | Animal pathogen | NCBI |
| *Aspergillus nidulans* | Pezizomycotina; Eurotiomycetes | 2 | 2, 8 | Saprotroph | NCBI |
| *Aspergillus niger* | Pezizomycotina; Eurotiomycetes | 5 | 2, 4, 7, 8(2) | Saprotroph | NCBI |
| *Aspergillus oryzae* | Pezizomycotina; Eurotiomycetes | 3 | 1, 7, 8 | Saprotroph | NCBI |
| *Aspergillus terreus* | Pezizomycotina; Eurotiomycetes | 6 | 1, 5, 7(2), 8, 9 | Animal pathogen | NCBI |
| *Botryotinia fuckeliana* | Pezizomycotina; Leotiomycetes | 2 | 1, 4 | Plant pathogen | Broad |
| *Candida albicans* | Saccharomycotina; Saccharomycetes | 0 | 0 | Animal pathogen | NCBI |
| *Candida dubliniensis* | Saccharomycotina; Saccharomycetes | 0 | 0 | Animal pathogen | Sanger |
| *Candida glabrata* | Saccharomycotina; Saccharomycetes | 0 | 0 | Animal pathogen | NCBI |
| *Candida guilliermondii* | Saccharomycotina; Saccharomycetes | 1 | 1 | Animal pathogen | Broad |
| *Candida parapsilosis* | Saccharomycotina; Saccharomycetes | 0 | 0 | Animal pathogen | Sanger |
| *Candida tropicalis* | Saccharomycotina; Saccharomycetes | 0 | 0 | Animal pathogen | NCBI |
| *Chaetomium globosum* | Pezizomycotina; Sordariomycetes | 1 | 1 | Plant pathogen | Broad |
| *Clavispora* (*Candida) lusitaniae* | Saccharomycotina; Saccharomycetes | 0 | 0 | Animal pathogen | Broad |
| *Coccidioides immitis* | Pezizomycotina; Eurotiomycetes | 0 | 0 | Animal pathogen | NCBI |
| *Coccidioides posadasii* | Pezizomycotina; Eurotiomycetes | 0 | 0 | Animal pathogen | NCBI |
| *Debaryomyces hansenii* | Saccharomycotina; Saccharomycetes | 1 | 1 | Saprotroph | NCBI |
| *Epichloë festucae*^††^ | Pezizomycotina; Sordariomycetes | 2 | 1, unique | Endophyte | Univ. of Kentucky |
| *Fusarium oxysporum f. sp. lycopersici* | Pezizomycotina; Sordariomycetes | 12 | 1, 2, 3, 4(2), 5, 6(2), 7, 8(3) | Plant pathogen | Broad |
| *Fusarium verticillioides* | Pezizomycotina; Sordariomycetes | 7 | 1, 2, 3, 5, 7, 8(2) | Plant pathogen | Broad |
| *Gibberella zeae* | Pezizomycotina; Sordariomycetes | 5 | 1, 3, 5, 7, 8 | Plant pathogen | NCBI |
| *Kluyveromyces lactis* | Saccharomycotina; Saccharomycetes | 1 | 1 | Saprotroph | NCBI |
| *Kluyveromyces waltii* | Saccharomycotina; Saccharomycetes | 1 | 1 | Saprotroph | NCBI |
| *Lodderomyces elongisporus* | Saccharomycotina; Saccharomycetes | 0 | 0 | Saprotroph | NCBI |
| *Magnaporthe grisea* | Pezizomycotina; Sordariomycetes incertae sedis | 2 | 1, 9 | Plant pathogen | Broad |
| *Mycosphaerella fijiensis* | Pezizomycotina; Dothideomycetes | 4 | 1, 5, 8, 9 | Plant pathogen | JGI |
| *Mycosphaerella graminicola* | Pezizomycotina; Dothideomycetes | 4 | 1, 7, 8, 9 | Plant pathogen | JGI |
| *Nectria haematococca* | Pezizomycotina; Sordariomycetes | 6 | 5, 6, 7, 8(3) | Plant pathogen | JGI |
| *Neosartorya fischeri* | Pezizomycotina; Eurotiomycetes | 4 | 1, 2, 8 | Animal pathogen | TIGR |
| *Neurospora crassa* | Pezizomycotina; Sordariomycetes | 1 | 6 | Saprotroph | NCBI |
| *Paracoccidioides brasiliensis* | Pezizomycotina; Eurotiomycetes | 0 | 0 | Animal pathogen | Broad |
| *Penicillium marneffei* | Pezizomycotina; Eurotiomycetes | 0 | 0 | Animal pathogen | NCBI |
| *Pichia stipitis* | Saccharomycotina; Saccharomycetes | 0 | 0 | Saprotroph | JGI |
| *Pneumocystis carinii* | Taphrinomycotina; Pneumocystidomycetes | 0 | 0 | Animal pathogen | Univ. of Cincinnati |
| *Podospora anserina* | Pezizomycotina; Sordariomycetes | 0 | 0 | Saprotroph | Univ. Paris Sud |
| *Pyrenophora tritici-repentis* | Pezizomycotina; Dothideomycetes | 3 | 1, 7, 9 | Plant pathogen | Broad |
| *Saccharomyces bayanus* | Saccharomycotina; Saccharomycetes | 1 | 1 | Saprotroph | NCBI |
| *Saccharomyces castellii* | Saccharomycotina; Saccharomycetes | 0 | 0 | Saprotroph | NCBI |
| *Saccharomyces cerevisiae* | Saccharomycotina; Saccharomycetes | 1 | 1 | Saprotroph | NCBI |
| *Saccharomyces kluyveri* | Saccharomycotina; Saccharomycetes | 3 | 1(3) | Saprotroph | NCBI |
| *Saccharomyces kudriavzevii* | Saccharomycotina; Saccharomycetes | 1 | 1 | Saprotroph | NCBI |
| *Saccharomyces mikatae* | Saccharomycotina; Saccharomycetes | 1 | 1 | Saprotroph | NCBI |
| *Saccharomyces paradoxus* | Saccharomycotina; Saccharomycetes | 1 | 1 | Saprotroph | NCBI |
| *Schizosaccharomyces japonicus* | Taphrinomycotina; Schizosaccharomycete | 1 | 1 | Saprotroph | NCBI |
| *Schizosaccharomyces pombe* | Taphrinomycotina; Schizosaccharomycetes | 2 | 1(2) | Saprotroph | NCBI |
| *Sclerotinia sclerotiorum* | Pezizomycotina; Leotiomycetes | 1 | 1 | Plant pathogen | Broad |
| *Stagonospora nodorum* | Pezizomycotina; Dothideomycetes | 4 | 1, 3, 5, 9 | Plant pathogen | Broad |
| *Talaromyces stipitatus* | Pezizomycotina; Eurotiomycetes | 5 | 2(2), 4, 8(2) | Saprotroph | NCBI |
| *Trichoderma atroviride* | Pezizomycotina; Sordariomycetes | 1 | 8 | Saprotroph/  Mycoparasite | NCBI |
| *Trichoderma reesei* | Pezizomycotina; Sordariomycetes | 0 | 0 | Saprotroph | JGI |
| *Trichoderma virens* | Pezizomycotina; Sordariomycetes | 1 | 8 | Saprotroph/  Mycoparasite | JGI |
| *Uncinocarpus reesii* | Pezizomycotina; Eurotiomycetes | 0 | 0 | Saprotroph | NCBI |
| *Vanderwaltozyma polyspora* | Saccharomycotina; Saccharomycetes | 2 | 1(2) | Saprotroph | NCBI |
| *Verticillium dahliae* | Pezizomycotina; Sordariomycetes | 3 | 6, 8,9 | Plant pathogen | Broad |
| *Yarrowia lipolytica* | Saccharomycotina; Saccharomycetes | 0* | 0 | Saprotroph | NCBI |
|  |  |  |  |  |  |
|  | **Phylum Basidiomycota** |  |  |  |  |
| *Coprinopsis cinereus* | Agaricomycotina; Agaricomycetes | 0 | 0 | Saprotroph | NCBI |
| *Cryptococcus neoformans* | Agaricomycotina; Tremellomycetes | 1 | 1 | Animal pathogen | NCBI |
| *Laccaria bicolor* | Agaricomycotina; Agaricomycetes | 0 | 0 | Mycorrhiza | JGI |
| *Malassezia globosa* | Ustilaginomycotina incertae sedis | 0 | 0 | Animal pathogen | Broad |
| *Phanerochaete chrysosporium* | Agaricomycotina; Agaricomycetes | 0 | 0 | Saprotroph | NCBI |
| *Postia placenta* | Agaricomycotina; Agaricomycetes | 0 | 0 | Saprotroph | JGI |
| *Puccinia graminis* | Pucciniomycotina; Pucciniomycetes | 2 | 7(2) | Plant pathogen | NCBI |
| *Sporobolomyces roseus*^†^ | Pucciniomycotina; Microbotryomycetes | 1 | unique | Saprotroph | JGI |
| *Ustilago maydis* | Ustilaginomycotina; Ustilaginomycetes | 2 | 1, 8 | Plant pathogen | NCBI |
|  |  |  |  |  |  |
|  | **Phylum Chytridiomycota** |  |  |  |  |
| *Batrachochytrium dendrobatidis* | Chytridiomycetes | 0 | 0 | Animal pathogen | Broad |
|  |  |  |  |  |  |
|  | **Phylum Microsporidia** |  |  |  |  |
| *Antonospora locustae* |  | 0 | 0 | Animal pathogen | Marine Bio. Lab. |
| *Encephalitozoon cuniculi* |  | 0 | 0 | Animal pathogen | NCBI |
|  |  |  |  |  |  |
|  | **Phylum Zygomycota** |  |  |  |  |
| *Phycomyces blakesleeanus*^†^ | Mucoromycotina; Mucorales | 1 | unique | Saprotroph | JGI |
| *Rhizopus oryzae* | Mucoromycotina; Mucorales | 0 | 0 | Saprotroph | Broad |

The GH32 clade number corresponds to the group numbers given for each of the well-supported clades labeled in Figure 1. If a genome contains more than one gene, each unique clade number is listed in ascending order, separated by commas. If more a genome contains more than one sequence in a given clade, the clade number is followed by the number of sequences found in that clade given in parentheses.

* *Yarrowia lipolytica* genome sequence contains GH32 gene SUC2 of *Saccharomyces cerevisiae* origin

^†^Unique sequence that does not belong to any designated clade
