# Supplementary material for: Friend or foe? Evolutionary history of glycoside hydrolase family 32 genes encoding for sucrolytic activity in fungi and its implications for plant-fungal symbioses
Source: BMC Evol Biol. 2009 Jun 30;9:148. doi: 10.1186/1471-2148-9-148 (PMC2728104; doi:10.1186/1471-2148-9-148)
Supplement: Additional file 2 — List of GH32 genes detected in fungal genomes. Table includes the number of GH32 genes detected, the GH32 clade to which each gene belongs, the accession number of each sequence, and the database from which they were retrieved. [file 1471-2148-9-148-S2.doc]

Additional file 2. List of GH32 genes detected in fungal genomes.

| **Organism** | **Classification** | **No. of GH32 genes** | **GH32 clade** | **GH32 Accession no.** | **Genome database** | **Comments** |
| --- | --- | --- | --- | --- | --- | --- |
|  |  |  |  |  |  |  |
|  | **Phylum Ascomycota** |  |  |  |  |  |
| *Alternaria brassicicola* | Pezizomycotina;Dothideomycetes | 2 | 7  9 | Contig5.80^2^  Cont1.185^2^ | Washington Univ. |  |
| *Ascosphaera apis* | Pezizomycotina; Eurotiomycetes | 4 | 1  5  5  5 | AARE01000788^2^  AARE01006445^2^  AARE01005240+5182^2^AARE01001923^2^ | USDA |  |
| *Ashbya gossypii* | Saccharomycotina;Saccharomycetes | 1 | 1 | NM212212^1^ | NCBI |  |
| *Aspergillus clavatus* | Pezizomycotina; Eurotiomycetes | 1 | 1 | XP001273181^1^ | NCBI |  |
| *Aspergillus flavus* | Pezizomycotina; Eurotiomycetes | 3 | 1  7  8 | AAIH02000210^2^  AAIH02000094^2^  AAIH02000012^2^ | TIGR | 5’ truncated |
| *Aspergillus fumigatus* | Pezizomycotina; Eurotiomycetes | 4 | 1  2  4  8 | XP_749260^1^  XP_748291^1^  XP_748286^1^  XP_747578^1^ | NCBI |  |
| *Aspergillus nidulans* | Pezizomycotina; Eurotiomycetes | 2 | 2  8 | EAA61090^3^  EAA59102^1^ | NCBI | Trimmed extra sequence |
| *Aspergillus niger* | Pezizomycotina; Eurotiomycetes | 5 | 2  4  7  8  8 | ABB59682^1^  ABB59681^1^  ABB59678^1^  ABB59679^1^  ABB59680^1^ | NCBI |  |
| *Aspergillus oryzae* | Pezizomycotina; Eurotiomycetes | 3 | 1  7  8 | XP001823245 ^1^  XP001824925^1^ XP001822932^1^  XP001826660^4^ | NCBI | Excluded |
| *Aspergillus terreus* | Pezizomycotina; Eurotiomycetes | 6 | 1  5  7  7  8  9 | XP001216776^1^  XP001210042^1^  XP001216100^1^  XP001214174^1^  XP001215038^1^  XP001218601^1^ | NCBI |  |
| *Botryotinia fuckeliana* | Pezizomycotina; Leotiomycetes | 2 | 1  4 | BC1G 10247.1^1^  BC1G 16010.1^3^ | Broad |  |
| *Candida guilliermondii* | Saccharomycotina; Saccharomycetes | 1 | 1 | PGUG 02777.1^1^ | Broad |  |
| *Chaetomium globosum* | Pezizomycotina; Sordariomycetes | 1 | 1 | CHGG 02287.1^1^  CHGG 07684.1^4^ | Broad | Excluded |
| *Debaryomyces hansenii* | Saccharomycotina; Saccharomycetes | 1 | 1 | XP461505^1^ | NCBI |  |
| *Epichloë festucae^††^* | Pezizomycotina; Sordariomycetes | 2 | 1  unique | contig 267^2^  contig 180^2^ | Univ. of Kentucky |  |
| *Fusarium oxysporum f. sp. lycopersici* | Pezizomycotina; Sordariomycetes | 12 | 1  2  3  4  4  5  6  6  7  8  8  8  -  - | FOXG 03944.2^1^  FOXG 11757.2^1^  FOXG 11756.2^1^  FOXG 15097.2^1^  FOXG 17513.2^1^  FOXG 04406.2^3^  FOXG 17514.2^3^  FOXG 14383.2^1^  FOXG 03211.2^1^  FOXG 17623.2^1^  FOXG 09916.2^1^  FOXG 17516.2^3^  FOXG 14385.2^4^  FOXG_15099.2^4^ | Broad | 5’ truncated  Excluded-Redundant  Excluded-Redundant |
| *Fusarium verticillioides* | Pezizomycotina; Sordariomycetes | 7 | 1  2  3  5  7  8  8 | FVEG 11933.3^3^  FVEG 10083.3^1^  FVEG 10082.3^1^  FVEG 07525.3^3^  FVEG 02083.3^1^  FVEG 12976.3^3^  FVEG 08584.3^1^ | Broad |  |
| *Gibberella zeae* | Pezizomycotina; Sordariomycetes | 5 | 1  3  5  7  8 | EAA69879^1^  EAA78236^1^  EAA69589^3^  EAA71892^1^  EAA72735^1^ | NCBI |  |
| *Kluyveromyces lactis* | Saccharomycotina; Saccharomycetes | 1 | 1 | XP451456^1^ | NCBI |  |
| *Kluyveromyces waltii* | Saccharomycotina; Saccharomycetes | 1 | 1 | AADM01000038+39^3^ | NCBI | Spanned 2 contigs |
| *Magnaporthe grisea* | Pezizomycotina; Sordariomycetes incertae sedis | 2 | 1  9  -  -  - | MGG 02507.5^1^  MGG 05785.5^1^  MGG 07837.5^4^  MGG 10748.5^4^  MGG 10767.4^4^ | Broad | Excluded  Excluded  Excluded |
| *Mycosphaerella fijiensis* | Pezizomycotina; Dothideomycetes | 4 | 1  5  8  9 | estExt Gw1.C30145^1^  estExt Gw1.1.321.1^1^  estExt Gw1.3.112.1^1^  estExt Gw1.C60340^1^ | JGI | 5’ truncated |
| *Mycosphaerella graminicola* | Pezizomycotina; Dothideomycetes | 4 | 1  7  8  9 | 63373^1^  38969^1^  55645^1^  42837^1^ | JGI | 5’ truncated |
| *Nectria haematococca* | Pezizomycotina; Sordariomycetes | 6 | 5  6  7  8  8  8 | 96948^1^  122843^1^  77788^1^  53858^1^  41174^1^  48604^1^ | JGI |  |
| *Neosartorya fischeri* | Pezizomycotina; Eurotiomycetes | 3 | 1  2  8 | NFIA 033540^1^  NFIA 042050^1^  NFIA 051560^1^  NF1A 041980^4^ | TIGR |  |
| *Neurospora crassa* | Pezizomycotina; Sordariomycetes | 1 | 6 | EAA32020^1^ | NCBI |  |
| *Pyrenophora tritici-repentis* | Pezizomycotina; Dothideomycetes | 3 | 1  7  9 | PTRG_10925.1^1^  PTRG_05210.1^1^  PTRG_06364.1^1^ | Broad |  |
| *Saccharomyces bayanus* | Saccharomycotina; Saccharomycetes | 1 | 1 | AACA01000015^2^ | NCBI |  |
| *Saccharomyces cerevisiae* | Saccharomycotina; Saccharomycetes | 1 | 1 | CAF29076^1^ | NCBI |  |
| *Saccharomyces kluyveri* | Saccharomycotina; Saccharomycetes | 3 | 1  1  1 | AACE03000007^2^  AACE03000004^2^  AACE03000002^2^ | NCBI |  |
| *Saccharomyces kudriavzevii* | Saccharomycotina; Saccharomycetes | 1 | 1 | AACI02000130^2^ | NCBI |  |
| *Saccharomyces mikatae* | Saccharomycotina; Saccharomycetes | 1 | 1 | AACH01000245^2^ | NCBI | 2 identical seqs in genbank 1 in Broad |
| *Saccharomyces paradoxus* | Saccharomycotina; Saccharomycetes | 1 | 1 | AABY01000004^2^ | NCBI |  |
| *Schizosaccharomyces japonicus* | Taphrinomycotina; Schizosaccharomycete | 1 | 1 | AATM01000114^2^ | NCBI |  |
| *Schizosaccharomyces pombe* | Taphrinomycotina; Schizosaccharomycetes | 2 | 1  1 | NP588300^1^  NP594168^1^ | NCBI |  |
| *Sclerotinia sclerotiorum* | Pezizomycotina; Leotiomycetes | 1 | 1 | SS1G 07184.1^1^ | Broad |  |
| *Stagonospora nodorum* | Pezizomycotina; Dothideomycetes | 4 | 1  3  5  9 | SNOG 11049.1^1^  SNOG 00610.1^1^  SNOG 01192.1^1^  SNOG 12253.1^1^ | Broad |  |
| *Talaromyces stipitatus* | Pezizomycotina; Eurotiomycetes | 5 | 2  2  4  8  8 | ABAS01000045.1a^2^  ABAS01000001.1^2^  ABAS01000045.1b^2^  ABAS01000002.1^2^  ABAS01000045.1c^2^ | NCBI |  |
| *Trichoderma atroviride* | Pezizomycotina; Sordariomycetes | 1 | 8 | ABDG01000249.1^2^ | NCBI |  |
| *Trichoderma virens* | Pezizomycotina; Sordariomycetes | 1 | 8 | 111987^1^ | JGI |  |
| *Vanderwaltozyma polyspora* | Saccharomycotina; Saccharomycetes | 2 | 1  1 | XP001642290^1^  XP001643480^1^ | NCBI |  |
| *Verticillium dahliae* | Pezizomycotina; Sordariomycetes | 3 | 6  8  9 | supercontig 4^2^  supercontig 10^2^  supercontig 6^2^ | Broad |  |
|  |  |  |  |  |  |  |
|  | **Phylum Basidiomycota** |  |  |  |  |  |
| *Cryptococcus neoformans* | Agaricomycotina; Tremellomycetes | 1 | 1 | XP567775^1^ | NCBI |  |
| *Puccinia graminis* | Pucciniomycotina; Pucciniomycetes | 2 | 7  7 | AAWC01000861^2^  AAWC01001393^2^ | NCBI |  |
| *Sporobolomyces roseus*^†^ | Pucciniomycotina; Microbotryomycetes | 1 | unique | 11505^1^ | JGI |  |
| *Ustilago maydis* | Ustilaginomycotina; Ustilaginomycetes | 2 | 1  8 | EAK82399^1^  EAK84508^1^ | NCBI |  |
|  |  |  |  |  |  |  |
|  | **Phylum Zygomycota** |  |  |  |  |  |
| *Phycomyces blakesleeanus*^†^ | Mucoromycotina; Mucorales | 1 | unique | 71917^1^ | JGI |  |
|  |  |  |  |  |  |  |
|  | **Other Eukaryotes** |  |  |  |  |  |
| *Arabidopsis thaliana* |  | 8 | -  -  -  -  -  -  -  - | NP_187994^1^  NP_564676^1^  NP_566464^1^  NP_190828^1^  NM_129177^1^  NP_568254^1^  NP_564798^1^  NP_563901^1^ | NCBI | OUTGROUP SEQUENCES |
|  |  |  |  |  |  |  |
|  | **Bacteria** |  |  |  |  |  |
| *Bacillus subtilis* |  | 3 |  | NP390581^1^  AF234992^1^  NP_391326^1^ | NCBI |  |
| *Escherichia coli* |  | 2 |  | YP_670282^1^  YP_670643^1^ | NCBI | Gene number varies among strains of *E. coli* |

GH32 gene number detected per fungal genome, the clade that each detected GH32 gene belonged to (Group number listed in Figure 3), and the Accession number or unique identifier corresponding to each GH32 gene sequence is listed.

^1^ Amino acid sequence predicted by model and retrieved by protein-protein blast.

^2^Amino acid sequence predicted manually.

^3^Either incorrect protein model prediction of amino acid sequence or complete sequence located in 2 different contigs and manually edited.

^4^Sequence similar to GH32 genes but were excluded from analysis because they were either: (1) unalignable to other sequences; (2) suspected of having incorrectable errors in protein prediction; (3) multiple model predictions for the same sequence.

^†^Unique sequence that does not belong to any designated clade.
