# Supplementary material for: Friend or foe? Evolutionary history of glycoside hydrolase family 32 genes encoding for sucrolytic activity in fungi and its implications for plant-fungal symbioses
Source: BMC Evol Biol. 2009 Jun 30;9:148. doi: 10.1186/1471-2148-9-148 (PMC2728104; doi:10.1186/1471-2148-9-148)
Supplement: Additional file 3 — List of fungal taxa tested for GH32 gene presence. The table includes the classification and ecological guild of each fungal taxon assayed for GH32 genes, whether or not they were detected, and the primer set(s) and PCR conditions used to assay each taxon. [file 1471-2148-9-148-S3.doc]

Additional file 3. List of fungal taxa tested for GH32 gene presence.

| **Organism** | **Strain** | **Classification** | **Ecological guild** | **GH32 detected** | **Primer set*** |
| --- | --- | --- | --- | --- | --- |
|  |  |  |  |  |  |
|  |  | **Phylum Ascomycota** |  |  |  |
| *Acarospora fuscata* | AcFu_TRM1209_TRM_PS1 | Lecanoromycetes; Acarosporales | Lichen | + | 1-1f/1-1r  1-1f/1-B1r |
| *Acremonium strictum* | olrim798 | Sordariomycetes; Hypocreales | Saprotroph | + | 1-1f/1-B1r |
| *Arthonia cinnabarina* | ArCi_GBP0182_TRM_PS1 | Arthoniomycetes; Arthoniales | Lichen | + | 1-1f/1-B1r |
| *Aureobasidium pullulans* | olrim951 | Incertae sedis | Plant pathogen | +^c^ | 1-1f/1-1r |
| *Baeomyces rufus* | BaRu_TRM1350_TRM_PS1 | Lecanoromycetes; Baeomycetales | Lichen | - |  |
| *Capnodium* sp. | olrim506 | Dothideomycetes; Capnodiales | Plant pathogen | + | 1-1f/1-1r |
| *Capronia munkii* | CBS615.96 | Eurotiomycetes; Chaetothyriales | Saprotroph | - |  |
| *Cenococcum geophilum* | UP162 | Dothideomycetes; Incertae sedis | Mycorrhizal | - |  |
| *Chalara* sp. | olrim396 | Leotiomycetes; Helotiales | Plant pathogen | - |  |
| *Chaunopycnis pustulata* | olrim66 | Sordariomycetes; Hypocreales | Endophyte | - |  |
| *Cladonia grayi* | DA_s.n., DUKE 0038797 | Lecanoromycetes; Lecanorales | Lichen | + | 1-1f/1-1r  1-1f/1-B1r  1-b1f/1-2.1r |
| *Cordyceps sinensis* | olrim985 | Sordariomycetes; Hypocreales | Animal pathogen | - |  |
| *Daldinia loculata* | olrim962 | Sordariomycetes; Xylariales | Endophyte/Saprotroph | - |  |
| *Diatrype undulata* | olrim324 | Sordariomycetes; Xylariales | Saprotroph | - |  |
| *Elaphomyces* sp. | AT2007032 | Eurotiomycetes; Eurotiales | Mycorrhizal | +^c^ | 5a-1f/5a-1r |
| *Epicoccum nigrum* | olrim796 | Dothideomycetes; Pleosporales | Saprotroph | + | 1-1f/1-1r |
| *Gnomonia gnomon* | CBS116384 | Sordariomycetes; Diaporthales | Plant pathogen | + | 1-1f/1-1r  1-1f/1-2.1r  1-1f/1-b1r |
| *Graphis scripta* | GrSc_TRM1361_TRM_PS1 | Lecanoromycetes; Ostropales | Lichen | - |  |
| *Hypocrea citrina* | olrim966 | Sordariomycetes; Hypocreales | Saprotroph | - |  |
| *Hypoxylon multiforme* | katrim1121 | Sordariomycetes; Xylariales | Endophyte | - |  |
| *Hypoxylon serpens* | olrim28 | Sordariomycetes; Xylariales | Endophyte | - |  |
| *Lachnum* sp. | olrim977 | Leotiomycetes; Helotiales | Endophyte | + | 1-1f/1-b1r |
| *Lecythophora hoffmannii* | olrim14 | Sordariomycetes; Incertae sedis | Saprotroph | - |  |
| *Lecythophora* sp. | olrim22 | Sordariomycetes; Incertae sedis | Saprotroph | + | 1-1f/1-2.1r |
| Leotiomycete | CBS486.95 | Leotiomycetes; Incertae sedis | Saprotroph | + | 1-1f/1-1r |
| *Leptodontidium orchidicola* | aurim621 | Leotiomycetes; Helotiales | Endophyte | + | 1-1f/1-1r |
| *Lophodermium piceae* | SLU26 | Leotiomycetes; Rhytismatales | Endophyte | + | 1-1f/1-1r |
| *Meliniomyces bicolor* | UP526 | Leotiomycetes; Incertae sedis | Mycorrhizal/Endophyte | + | 1-1f/1-1r  1-1f/1-b1r |
| *Ocellularia cf. minuta* | Ertz 9384, AFToL 2096 | Lecanoromycetes; Ostropales | Lichen | - |  |
| *Oidiodendron echinulatum* | aurim610 | Dothideomycetes; Incertae sedis | Endophyte | - |  |
| *Orbilia vinosa* | CBS917.72 | Orbiliomycetes; Orbiliales | Saprotroph | - |  |
| *Phialocephala fortinii* | V12 | Leotiomycetes; Helotiales | Endophyte | + | 1-1f/1-b1r |
| *Phialocephala* sp. | aurim1069 | Leotiomycetes; Helotiales | Endophyte | + | 1-1f/1-b1r |
| *Phialophora fastigiata* | olrim191 | Leotiomycetes; Helotiales | Endophyte | + | 1-1f/1-b1r |
| *Phialophora finlandica* | olrim217 | Leotiomycetes; Helotiales | Mycorrhizal/Endophyte | + | 1-1f/1-1r |
| *Phialophora malorum* | olrim128 | Leotiomycetes; Helotiales | Endophyte | + | 1-1f/1-1r |
| *Rhizina undulata* | olrim818 | Pezizomycetes; Pezizales | Plant pathogen | - |  |
| *Rhizoscyphus ericae* | UP505 | Leotiomycetes; Helotiales | Mycorrhizal | +  +^a^ | 1-1f/1-1r  6-1f/6-2r |
| *Sarcographa labyrinthica* | Ertz 9578, AFToL 2215 | Lecanoromycetes; Ostropales | Lichen | - |  |
| *Sarea difformis* | olrim45 | Lecanoromycetes; Agyriales | Saprotroph/Endophyte | - |  |
| *Sarea resinae* | olrim42 | Lecanoromycetes; Agyriales | Saprotroph/Endophyte | - |  |
| *Scorias spongiosa* | HR121 | Dothideomycetes; Capnodiales | Endophyte | - |  |
| *Taphrina deformans* | CBS102167 | Taphrinomycetes; Taphrinales | Saprotroph | + | 1-1f/1-2.1r |
| *Trichoderma lignorum* | SLU180 | Sordariomycetes; Hypocreales | Plant pathogen | - |  |
| *Tuber borchii* | GB33 | Pezizomycetes; Pezizales | Saprotroph | - |  |
| *Wilcoxina* sp. | aurim706 | Pezizomycetes; Pezizales | Mycorrhizal | - |  |
|  |  |  |  |  |  |
|  |  | **Phylum Basidiomycota** |  |  |  |
| *Agaricus bisporus* | ICA | Agaricomycetes; Agaricales | Saprotroph | - |  |
| *Amanita spissa* | AT2007034 | Agaricomycetes; Agaricales | Mycorrhizal | - |  |
| *Amylostereum chailletii* | SLU44 | Agaricomycetes; Russulales | Saprotroph | - |  |
| *Antrodia serialis* | olrim282 | Agaricomycetes; Polyporales | Saprotroph | - |  |
| *Armillaria borealis* | olrim1084 | Agaricomycetes; Agaricales | Plant pathogen/Saprotroph | - |  |
| *Armillaria mellea* | olrim1076 | Agaricomycetes; Agaricales | Saprotroph | - |  |
| *Bjerkandera adusta* | SLU43 | Agaricomycetes; Polyporales | Saprotroph | - |  |
| *Boletus aestivalis* | AT2007033 | Agaricomycetes; Boletales | Mycorrhizal | - |  |
| *Boletus edulis* | AT2007050 | Agaricomycetes; Boletales | Mycorrhizal | - |  |
| *Boletus luridiformis* | AT2007041 | Agaricomycetes; Boletales | Mycorrhizal | - |  |
| *Cantharellus cibarius* | AT2007027 | Agaricomycetes; Cantharellales | Mycorrhizal | - |  |
| *Cantharellus pallens* | AT2007037 | Agaricomycetes; Cantharellales | Mycorrhizal | - |  |
| *Ceratobasidium* sp. | olrim257 | Agaricomycetes; Ceratobasidiales | Saprotroph | - |  |
| *Ceriporiopsis* sp. | olrim4 | Agaricomycetes; Polyporales | Saprotroph | - |  |
| *Chalciporus piperatus* | AT2007061 | Agaricomycetes; Boletales | Mycorrhizal | - |  |
| *Chondrostereum purpureum* | olrim813 | Agaricomycetes; Polyporales | Plant pathogen | - |  |
| *Chroogomphus rutilus* | AT2007060 | Agaricomycetes; Boletales | Mycorrhizal | - |  |
| *Coltricia perennis* | CBS101387 | Agaricomycetes; Hymenochaetales | Mycorrhizal | - |  |
| *Coniophora puteana* | SLU50 | Agaricomycetes; Boletales | Saprotroph | - |  |
| *Cortinarius balteatocumatillus* | AT2007051 | Agaricomycetes; Agaricales | Mycorrhizal | - |  |
| *Cortinarius diasemospermus* | AT2007030 | Agaricomycetes; Agaricales | Mycorrhizal | - |  |
| *Cylindrobasidium laeve* | SLU53 | Agaricomycetes; Polyporales | Saprotroph | - |  |
| *Dacrymyces palmatus* | CBS196.63 | Dacrymycetes; Dacrymycetales | Saprotroph | - |  |
| *Daedalea quercina* | SLU59 | Agaricomycetes; Polyporales | Saprotroph | - |  |
| *Exidia pithya* | olrim482 | Agaricomycetes; Auriculariales | Plant pathogen | + | 1-1f/1-1r |
| *Fomitopsis pinicola* | SLU54 | Agaricomycetes; Polyporales | Saprotroph | +^d^ | 1-1f/1-1r |
| *Ganoderma applanatum* | olrim925 | Agaricomycetes; Polyporales | Saprotroph | - |  |
| *Gloeocystidiellum ochraceum* | olrim399 | Agaricomycetes; Russulales | Saprotroph | - |  |
| *Gloeophyllum cf. odoratum* | SLU75 | Agaricomycetes; Polyporales | Saprotroph | - |  |
| *Gloeophyllum sepiarium* | olrim1096 | Agaricomycetes; Polyporales | Saprotroph | + | 1-1f/1-1r  1-1f/1-b1r |
| *Gloeoporus taxicola* | olrim879 | Agaricomycetes; Polyporales | Saprotroph | - |  |
| *Gomphidius maculatus* | AT2007060 | Agaricomycetes; Boletales | Mycorrhizal | - |  |
| *Hapalopilus croceus* | olrim823 | Agaricomycetes; Polyporales | Saprotroph/Plant pathogen | - |  |
| *Hebeloma crustuliniforme* | UP184 | Agaricomycetes; Agaricales | Mycorrhizal | - |  |
| *Heterobasidion annosum* | 95191 | Agaricomycetes; Russulales | Plant pathogen | - |  |
| *Hydnum repandum* | AT2007048 | Agaricomycetes; Cantharellales | Mycorrhizal | - |  |
| *Hygrophorus cf. agathosmus* | AT2007001 | Agaricomycetes; Agaricales | Mycorrhizal | - |  |
| *Hymenochaete tabacina* | katrim1132 | Agaricomycetes; Hymenochaetales | Saprotroph | - |  |
| *Inocybe cinncinnata* | AT2007031 | Agaricomycetes; Agaricales | Mycorrhizal | - |  |
| *Inonotus radiatus* | olrim69 | Agaricomycetes; Hymenochaetales | Saprotroph | - |  |
| *Ischnoderma benzoinum* | katrim1102 | Agaricomycetes; Polyporales | Saprotroph | - |  |
| *Lactarius fulvissimus* | CN0701 | Agaricomycetes; Russulales | Mycorrhizal | - |  |
| *Lactarius glyciosmus* | UP559 | Agaricomycetes; Russulales | Mycorrhizal | - |  |
| *Lactarius pubescens* | CN0740 | Agaricomycetes; Russulales | Mycorrhizal | - |  |
| *Lactarius quieticolor* | CN0739 | Agaricomycetes; Russulales | Mycorrhizal | - |  |
| *Lactarius rufus* | CN0722 | Agaricomycetes; Russulales | Mycorrhizal | - |  |
| *Lactarius scrobiculatus* | CN0702 | Agaricomycetes; Russulales | Mycorrhizal | - |  |
| *Laetiporus sulphureus* | olrim118 | Agaricomycetes; Polyporales | Saprotroph/Plant pathogen | - |  |
| *Lentinellus omphaloides* | olrim297 | Agaricomycetes; Russulales | Saprotroph | - |  |
| *Lenzites betulina* | SLU58 | Agaricomycetes; Polyporales | Saprotroph | + | 1-1f/1-1r |
| *Merulius tremellosus* | SLU45 | Agaricomycetes; Polyporales | Saprotroph | - |  |
| *Mycoacia uda* | olrim251 | Agaricomycetes; Polyporales | Saprotroph | - |  |
| *Neolentinus lepideus* | SLU33 | Agaricomycetes; Polyporales | Saprotroph | + | 1-1f/1-1r  1-1f/1-b1r |
| *Paxillus filamentosus* | AT2007039 | Agaricomycetes; Boletales | Mycorrhizal | - |  |
| *Paxillus involutus* | AT2001015 | Agaricomycetes; Boletales | Mycorrhizal | - |  |
| *Peniophora cinerea* | olrim226 | Agaricomycetes; Russulales | Saprotroph | + | 1-1f/1-1r |
| *Peniophora incarnata* | olrim771 | Agaricomycetes; Russulales | Saprotroph | + | 1-1f/1-1r  1-1f/1-2.1r |
| *Peniophora piceae* | olrim10 | Agaricomycetes; Russulales | Saprotroph | + | 1-1f/1-1r |
| *Phaeolus schweinitzii* | olrim219 | Agaricomycetes; Polyporales | Plant pathogen/Saprotroph | - |  |
| *Phanerochaete chrysosporium* | MK/SLU | Agaricomycetes; Corticiales | Saprotroph | - |  |
| *Phellinus robustus* | olrim122 | Agaricomycetes; Hymenochaetales | Plant pathogen | - |  |
| *Phellinus tremulae* | SLU34 | Agaricomycetes; Hymenochaetales | Plant pathogen | - |  |
| *Phlebia centrifuga* | olrim220 | Agaricomycetes; Polyporales | Saprotroph | - |  |
| *Phlebia radiata* | olrim344 | Agaricomycetes; Polyporales | Saprotroph | - |  |
| *Phlebia ruta* | olrim921 | Agaricomycetes; Polyporales | Saprotroph | - |  |
| *Pholiota squarrosa* | SLU62 | Agaricomycetes; Agaricales | Saprotroph | + | 1-1f/1-b1r |
| *Piloderma byssinum* | UP582 | Agaricomycetes; Atheliales | Mycorrhizal | - |  |
| *Piloderma fallax* | UP581 | Agaricomycetes; Atheliales | Mycorrhizal | - |  |
| *Piloderma olivaceum* | JLPCC08 | Agaricomycetes; Atheliales | Mycorrhizal | - |  |
| *Piptoporus betulinus* | olrim137 | Agaricomycetes; Polyporales | Saprotroph | - |  |
| *Polyporus brumalis* | olrim764 | Agaricomycetes; Polyporales | Saprotroph | + | 1-1f/1-1r |
| *Pseudocratarellus undulatus* | AT2007047 | Agaricomycetes; Cantharellales | Mycorrhizal | - |  |
| *Pycnoporus cinnabarinus* | SLU66 | Agaricomycetes; Polyporales | Saprotroph | + | 1-1f/1-1r |
| *Ramaria gracilis* | CBS150.74 | Agaricomycetes; Gomphales | Mycorrhizal | - |  |
| *Rhizoctonia solani* | SLU167 | Agaricomycetes; Incertae sedis | Plant pathogen | - |  |
| *Rhizopogon luteolus* | AT2007049 | Agaricomycetes; Boletales | Mycorrhizal | - |  |
| *Russula albonigra* | AT2007026 | Agaricomycetes; Russulales | Mycorrhizal | - |  |
| *Russula aurea* | AT2007036 | Agaricomycetes; Russulales | Mycorrhizal | - |  |
| *Russula caerulea* | AT2007025 | Agaricomycetes; Russulales | Mycorrhizal | - |  |
| *Russula chloroides* | UP528 | Agaricomycetes; Russulales | Mycorrhizal | - |  |
| *Russula cremeoavellanea* | AT2007038 | Agaricomycetes; Russulales | Mycorrhizal | - |  |
| *Russula foetens* | AT2007040 | Agaricomycetes; Russulales | Mycorrhizal | - |  |
| *Russula font-queri* | AT2007029 | Agaricomycetes; Russulales | Mycorrhizal | - |  |
| *Russula vesca* | AT2007043 | Agaricomycetes; Russulales | Mycorrhizal | - |  |
| *Russula xerampelina* | AT2007035 | Agaricomycetes; Russulales | Mycorrhizal | - |  |
| *Sarcodon imbricatus* | UP590 | Agaricomycetes; Thelephorales | Mycorrhizal | - |  |
| *Schizophyllum commune* | olrim499 | Agaricomycetes; Agaricales | Saprotroph | + | 1-1f/1-1r  1-1f/1-2.1r |
| *Sebacina incrustans* | AT2007058 | Agaricomycetes; Sebacinales | Mycorrhizal | + | 1-1f/1-1r  1-b1f/1-2.1r |
| *Serpula lacrymans* | NH/SLU | Agaricomycetes; Boletales | Saprotroph | - |  |
| *Sparassis crispa* | olrim1012 | Agaricomycetes; Polyporales | Plant pathogen/Saprotroph | - |  |
| *Stereum hirsutum* | SLU39 | Agaricomycetes; Russulales | Saprotroph | + | 1-1f/1-1r  1-b1f/1-2.1r |
| *Stereum sanguinolentum* | olrim881 | Agaricomycetes; Russulales | Saprotroph | + | 1-1f/1-2.1r |
| *Suillus granulatus* | AT2007042 | Agaricomycetes; Boletales | Mycorrhizal | - |  |
| *Suillus variegatus* | AT1998114 | Agaricomycetes; Boletales | Mycorrhizal | - |  |
| *Thelephora terrestris* | TU10086 | Agaricomycetes; Thelephorales | Mycorrhizal | - |  |
| *Tilletia caries* | CBS160.85 | Exobasidiomycetes; Tilletiales | Plant pathogen | - |  |
| *Tomentellopsis cf. stuposa* | UP610 | Agaricomycetes; Thelephorales | Mycorrhizal | - |  |
| *Trametes hirsuta* | katrim1131 | Agaricomycetes; Polyporales | Saprotroph | + | 1-1f/1-1r |
| *Trametes versicolor* | olrim882 | Agaricomycetes; Polyporales | Saprotroph | + | 1-1f/1-1r |
| *Trametes zonata* | katrim1105 | Agaricomycetes; Polyporales | Saprotroph | - |  |
| *Tremella fuciformis* | CBS8226 | Tremellomycetes; Tremellales | Saprotroph | - |  |
| *Tyromyces chioreus* | katrim1130 | Agaricomycetes; Polyporales | Saprotroph | - |  |
| *Uloporus lividus* | UP179 | Agaricomycetes; Boletales | Mycorrhizal | - |  |

The symbols + and - indicate whether GH32 genes were or were not detected, respectively. All GH32 genes detected in this study were members of group 1 (see Figure 3) except those labelled with a lettered subscript beside the + symbol. All primer pair combinations that successfully amplified GH32 genes for a given taxon and the PCR conditions used for each combination are given.

^a^ Group 5 GH32 sequence detected

^b^ Group 6 GH32 sequence detected

^c^ Group 8 GH32 sequence detected

^d^ Group 9 GH32 sequence detected

*Annealing temperatures used in PCR amplification for primer sets: 1-1f/1-1r, 1-1f/1-b1r = 45º C; 1-b1f/1-2r, 4-2f/4-1r, 5a-1f/5a-1r, 5b-a1f/5b-1.1r = 50º C; 2-1f/2-2r, 5b-b1f/5b-1.5r, 5c-1f/5c-1r, 6-1f/6-2r = 55º C; 1-1f/1-2.1r, 1-b1f/1-2.1r = 56º C
