# Supplementary material for: Friend or foe? Evolutionary history of glycoside hydrolase family 32 genes encoding for sucrolytic activity in fungi and its implications for plant-fungal symbioses
Source: BMC Evol Biol. 2009 Jun 30;9:148. doi: 10.1186/1471-2148-9-148 (PMC2728104; doi:10.1186/1471-2148-9-148)
Supplement: Additional file 4 — List of fungal genome RNA polymerase accession numbers. This table includes information for the data used for phylogenetic reconstruction of fungi with completely sequenced genomes. [file 1471-2148-9-148-S4.doc]

Additional file 4. RNA polymerase accession numbers used for phylogenetic reconstruction of fungi with completely sequenced genomes.

| **Organism** | **RNA Polymerase Gene Accession Numbers**  **RPA1 RPA2 RPB1 RPB2 RPC1 RPC2** | | | | | | **Data-base** |
| --- | --- | --- | --- | --- | --- | --- | --- |
| *Ajellomyces capsulatus* | XP001539109 | XP001538304 | XP001542193 | XP001540601 | XP001536231 | XP001539366 | NCBI |
| *Ajellomyces dermatitidis* | Contig 9^1^ | Contig 150 | Contig 43^2^ | Contig 406 | Contig 29^1^ | Contig 242 | WA Univ. |
| *Alternaria brassicicola* | Contig 10.38^2^ | Contig 14.61 | Contig 2.147^2^ | Contig 2.243 | Contig 7.42 | Contig 5.199^1^ | WA Univ. |
| *Antonospora locustae* | AAT12325^2^ | Contig19 | AF061288 | Contig543 | orf:2548 | AAT12334 | NCBI/ Marine Bio Lab. |
| *Arabidopsis thaliana* | NP191325 | NP564341 | NP195305.1 | NP193902 | NP200812 | NP199327.4 | NCBI |
| *Ascosphaera apis* | AARE01005892  AARE01001695  AARE01001292 | AARE01000010 | AARE01003288^2^  AARE01005530 | AARE01003556^2^  AARE01006306 | AARE01005163^2^ | AARE01002708^2^  AARE01001951 | NCBI |
| *Ashbya gossypii* | NP984470 | NP982975 | NP984182 | NP985951 | NP985109 | NP983821 | NCBI |
| *Aspergillus clavatus* | XP001268868 | XP001267972 | XP001268791 | XP001272355 | XP001271388 | XP001269884 | NCBI |
| *Aspergillus flavus* | AAIH02000190 | AAIH02000245 | AAIH02000051 | AAIH02000001.1 | AAIH02000094 | AAIH02000331 | NCBI |
| *Aspergillus fumigatus* | XP752760.1 | XP750719.1 | XP752837 | XP746740 | XP754189.1 | XP749996.1 | NCBI |
| *Aspergillus nidulans* | EAA63997^1,2^ | EAA59242 | EAA65639 | EAA61953 | EAA63997 | EAA65727 | NCBI |
| *Aspergillus niger* | XP001392820 | XP001394181 | XP001389676 | XP001395161 | XP001393726 | XP001388998 | NCBI |
| *Aspergillus oryzae* | XP001727495 | XP001819329 | XP001819667 | XP001825469 | XP001824913 | XP001817863 | NCBI |
| *Aspergillus terreus* | XP001210679.1 | XP001215005 | XP001210766 | XP001209185 | XP001212669 | XP001214023 | NCBI |
| *Batrachochytrium dendrobatidis* | BDEG03907.1 | BDEG04902.1 | BDEG04054.1^2^ | BDEG06085.1 | BDEG03112.1 | BDEG03933.1 | Broad |
| *Botryotinia fuckeliana* | XP001548665^2^ | XP001546494 | XP001549037 | XP001552822 | XP001550869 | NA | NCBI |
| *Candida albicans* | EAK91475 | XP888689 | EAL00529 | EAK99240 | EAK96387 | EAK91554 | NCBI |
| *Candida dubliniensis* | CHR1.070111 | CHR7.070112 | CHRR.070112 | CHR1.070111 | CHR4.070111 | CHRR.070112 | Sanger |
| *Candida glabrata* | XP445928 | XP447785.1 | XP447415 | XM448959 | XP449275 | XP448895 | NCBI |
| *Candida guilliermondii* | PGUG03709.1 | PGUG00409.1 | A5DCV30^1^* | PGUG02833.1 | PGUG04343.1 | PGUG04096.1 | NCBI/ Broad |
| *Candida parapsilosis* | CPAG02639.0 | CPAG03894.0 | CPAG00258. | CPAG00865.0 | CPAG01415.0 | CPAG04095.0 | Broad |
| *Candida tropicalis* | AAFN01000128.1^1^ | CTRG05038.3 | AAFN01000119^1,2^ | CTRG04456.3 | CTRG05772.3^2^ | CTRG00585.3 | NCBI/ Broad |
| *Chaetomium globosum* | XP001228221^2^ | XP001220654^2^ | XP001220925 | XP001226434 | XP001226555 | XP001225506 | NCBI |
| *Clavispora* (*Candida) lusitaniae* | AAFT01000032^1^ | CLUG03112.1 | CLUG03468.1^1^ | AAFT01000003 | CLUG02993.1 | CLUG02458.1 | NCBI/ Broad |
| *Coccidioides immitis* | XP001243937 | XP001247351 | XP001243803 | XP001240650 | XP001246195 | XP001242390 | NCBI |
| *Coccidioides posadasii* | CIMG03378.3 | CIMG01122.3 | CIMG03244.3 | CIMG07813.3 | CIMG05636.3 | CIMG06286.3 | Broad |
| *Cryptococcus neoformans* | XP570516 | XP571380 | XP570943 | XP570204 | XP571468 | XP572718 | NCBI |
| *Debaryomyces hansenii* | XP458147 | XP461338 | XP456921 | NC006049^2†^ | XP462102 | XP462552 | NCBI |
| *Encephalitozoon cuniculi* | NP584825 | NP597555 | NP597540 | NP586140 | NP585937 | NP586343 | NCBI |
| *Epichloë festucae* | Contig283 | Contig550 | Contig71 | Contig180 | Contig132^1^ | Contig419^1^ | Univ. KY |
| *Fusarium oxysporum f. sp. lycopersici* | FOXG13951.2 | FOXG09260.2 | FOXG00887.2 | FOXG10639.2 | NA | FOXG00800.2 | Broad |
| *Fusarium verticillioides* | FVEG11373.3 | FVEG06860.3 | FVEG00683.3 | FVEG09286.3 | FVEG02570.3 | FVEG00714.3 | Broad |
| *Gibberella zeae* | XP384467 | XP385803 | XP381092 | NA | XP390036 | XP381019 | NCBI |
| *Kluyveromyces lactis* | XP456115 | XP451816 | XP455310 | XP451784 | XP454912 | XP455128 | NCBI |
| *Kluyveromyces waltii* | AADM01000080 | AADM01000303 | AADM01000294 | AADM01000162 | AADM01000254^1^ | AADM01000104 | NCBI |
| *Laccaria bicolor* | XP001876601 | XP001877002 | XP001881394 | NZABFE01000008^1^ | XP001884255 | XP001880038 | NCBI |
| *Lodderomyces elongisporus* | XP001527964 | XP001523265 | XP001523387 | XP001526602 | XP001523103 | XP001525350 | NCBI |
| *Magnaporthe grisea* | XP368086 | XP363646 | XP362207 | XP362269 | XP362032 | XP370487 | NCBI |
| *Malassezia globosa* | XP001728958^2^ | XP001730112^1§^  XP001730113 | XP001729796 | XP001731621 | XP001730008 | XP001731706 | NCBI |
| *Monosiga brevicollis* | Monbr1 16642^2^ | Monbr1 12630 | Monbr1 38135 | Monbr1 15585^2^ | Monbr1 39165 | Monbr1 26625 | JGI |
| *Mycosphaerella fijiensis* | fgenesh1pm.Cscaffold12000041 | estExtGenewise1Plus.C120263 | estExtGenewise1Plus.C90438 | estExtGenewise1Plus.C90161 | fgenesh1pg.Cscaffold9000015 | egw1.16.645.1^2^ | JGI |
| *Mycosphaerella graminicola* | estExtfgenesh2pg.Csca4000003 | estExtfgenesh2pg.C40383 | estExtfgenesh2pg.C50297 | estExtfgenesh2pg.C50243 | estExtfgenesh2pg.C11669 | egw.2.1184.1^2^ | JGI |
| *Nectria haematococca* | estExtGw1.11.400.1 | fgenesh1pm.sca10chr820000088 | estExtfgenesh1pm.Csca1chr1300437 | estExtGw1Plus.Csca20chr6401146 | estExtGgw1.5.8.1 | fgenesh1pm.sca1chr130000384 | JGI |
| *Neosartorya fischeri* | NFIA011570 | NFIA053940 | NFIA010730 | NFIA114650 | NFIA062940 | NFIA022140 | TIGR |
| *Neurospora crassa* | EAA26770 | EAA35588 | EAA34861 | EAA27870 | EAA27335 | EAA27959 | NCBI |
| *Paracoccidioides brasiliensis* | PAAG02197.1 | PAAG01151.1 | PAAG05397.1 | PAAG06854.1 | PAAG03412.1 | PAAG08071.1 | Broad |
| *Penicillium marneffei* | ABAR01000008 | ABAR01000019 | ABAR01000019 | ABAR01000057 | ABAR01000005 | ABAR01000005 | NCBI |
| *Phanerochaete chrysosporium* | Phchr1 130147 | Phchr1 131576 | Phchr1 127142 | Phchr1 7545 | Phchr1 129507 | Phchr1 121905 | JGI |
| *Phycomyces blakesleeanus* | Phybl1 74623 | Phybl1 74571 | Phybl1 57416^2^ | Phybl1 39603 | Phybl1 30889 | Phybl1 68328 | JGI |
| *Pichia stipitis* | XP001386054 | XP001384965 | EAZ62973 | EAZ63343 | EAZ63664 | XP001383024 | NCBI |
| *Pneumocystis carinii* | cap3it0APAssb072105.fasta.Contig7085^2^ | cap3it1grp346Contig249.268^2^ | cap3it1grp346Contig136 | cap3it1grp346Contig456^2^ | cap3it1grp346Contig518  cap3it0APAssb072105.fasta.Contig5868^1,2^ | cap3it0APAssb072105.fasta.Contig6093^2^ | Univ. of Cincinnati |
| *Podospora anserina* | Pa65360 | Pa18070 | Pa12650 | Pa41700 | Pa42310 | Pa37790 | Univ. Paris Sud |
| *Postia placenta* | estExtGenewise1Plus.C170127 | egw1.91.2.1 | egw1.13.112.1^2^ | estExtGenewise1.C80199 | estExtGenewise1.C300131 | estExtfgenesh3pm.C240012 | JGI |
| *Puccinia graminis* | PGTG06293.2 | PGTG11170.2 | PGTG15953.2 | PGTG03411.2 ^2^ | PGTG01084.2 | PGTG07203.2 | Broad |
| *Pyrenophora tritici-repentis* | XP001932229 | XP001932339 | XP001934953 | XP001934943 | XP001932816 | XP001933815 | NCBI |
| *Rhizopus oryzae* | RO3G16519.1 | RO3G08030.1 | supercontig 3.5^2^ | RO3G13232.1 | RO3G12475.1 | RO3G12315.1 | Broad |
| *Saccharomyces bayanus* | SBAY24264 | SBAY25575 | AACA01000430.1^1,2^  AACA01000773 | SBAY23874 | AACA01000119.1^2^  AACA01000229.1 | AACA01000114.1^1,2^  AACA01000376 | NCBI/ Broad |
| *Saccharomyces castellii* | AACF01000220.1^2^ | AACF01000015 | AACF01000092 | AACF01000010 | AACF01000010 | AACF01000106 | NCBI |
| *Saccharomyces cerevisiae* | P10964.2 | P22138.1 | P04050.2 | P08518.2 | P04051.1 | P22276.2 | NCBI |
| *Saccharomyces kluyveri* | AACE03000007 | AACE03000007 | AACE03000002 | AACE03000001 | AACE03000001 | AACE03000006 | NCBI |
| *Saccharomyces kudriavzevii* | AACI02000046  AACI02000234 | AACI02000322  AACI02000358 | MISSING | AACI02000670  AACI02000805 | AACI02000301  AACI02000301 | AACI02001008/AACI02001507 | NCBI |
| *Saccharomyces mikatae* | SMIK20713 | SMIK22320 | AACH01000501  AABZ01000979.1^2^ | AABZ01000378.1^1,2^ | SMIK20242 | AABZ01000283^1,2^ | NCBI/ Broad |
| *Saccharomyces paradoxus* | SPAR21546 | SPAR23372 | AABY01000121.1^2^ | SPAR20881 | SPAR20768 | SPAR21099 | NCBI/ Broad |
| *Schizosaccharomyces japonicus* | SJAG03144.1^2^ | SJAG03578.1 | SJAG01713.1 | SJAG04030.1 | SJAG04934.1 | SJAG00637.1 | Broad |
| *Schizosaccharomyces pombe* | NP596300 | NP595819 | NP595673 | NP593101 | NP595506 | NP593690 | NCBI |
| *Sclerotinia sclerotiorum* | XP001589882 | XP001585849 | XP001593006 | XP001598796 | XP001588093 | XP001595664 | NCBI |
| *Sporobolomyces roseus* | Sporo1 16466 | Sporo1 34219 | Sporo1 14395 | Sporo1 22879 | Sporo1 28252 | Sporo1 19280 | JGI |
| *Stagonospora nodorum* | ABAS01000003 | ABAS01000007^1§^ | ABAS01000007 | ABAS01000030 | ABAS01000013 | ABAS01000011 | NCBI/ Broad |
| *Talaromyces stipitatus* | Triat1 54138 | Triat1 132650 | Triat1 89498 | Triat1 151043 | Triat1 86460 | Triat1 132468 | NCBI |
| *Trichoderma atroviride* | Trire2 6015 | Trire2 123220 | Trire2 79315 | Trire2 79225 | Trire2 76710 | Trire2 22496 | JGI |
| *Trichoderma reesei* | Trive1 57534 | Trive1 57191 | Trive1 85034 | Trive1 76818 | Trive1 28982 | Trive1 65285 | JGI |
| *Trichoderma virens* | UREG07206.1 | UREG01066.1 | UREG07095.1 | UREG02143.1 | UREG06114.1 | UREG04098.1 | JGI |
| *Uncinocarpus reesii* | ABAS01000003 | ABAS01000007 | ABAS01000007 | ABAS01000030^1^ | ABAS01000013 | ABAS01000011 | Broad |
| *Ustilago maydis* | EAK83798 | EAK81692 | EAK84769 | AAS67525.1^2^ | EAK87041 | EAK83484 | NCBI |
| *Vanderwaltozyma polyspora* | XP001643648 | XP001645652 | XP001642173 | XP001642238 | XP001643927 | XP001643385 | NCBI |
| *Verticillium dahliae* | Supercontig 30 | Supercontig 5 | Supercontig 13 | Supercontg 10 | Supercontig 14 | Supercontig 15 | Broad |
| *Yarrowia lipolytica* | XP505388 | XP503752 | XP501909 | XP502376 | XP502142 | XP500966 | NCBI |

* sequence spanned multiple contigs

^†^ error in original protein prediction; correct annotation unable to be discerned

^§^ error in original protein prediction causing sequence to be truncated and/or divided across multiple coding sequences

^1^ modified original protein prediction to correct error

^2^ partial sequence
